# Development of homozygous tetraploid potato and whole genome doubling-induced the enrichment of H3K27ac and potentially enhanced resistance to cold-induced sweetening in tubers

**DOI:** 10.1093/hr/uhad017

**Published:** 2023-02-08

**Authors:** Hongwei Guo, Min Zhou, Guoyan Zhang, Li He, Caihong Yan, Min Wan, Jianjun Hu, Wei He, Deying Zeng, Bo Zhu, Zixian Zeng

**Affiliations:** Department of Biological Science, College of Life Sciences, Sichuan Normal University, Chengdu 610101, Sichuan, China; Department of Biological Science, College of Life Sciences, Sichuan Normal University, Chengdu 610101, Sichuan, China; Department of Biological Science, College of Life Sciences, Sichuan Normal University, Chengdu 610101, Sichuan, China; Horticulture Institute, Sichuan Academy of Agricultural Sciences, Chengdu 610066, China; Horticulture Institute, Sichuan Academy of Agricultural Sciences, Chengdu 610066, China; Department of Biological Science, College of Life Sciences, Sichuan Normal University, Chengdu 610101, Sichuan, China; Department of Biological Science, College of Life Sciences, Sichuan Normal University, Chengdu 610101, Sichuan, China; Crop Research Institute, Sichuan Academy of Agricultural Sciences, Chengdu 610066, China; Crop Research Institute, Sichuan Academy of Agricultural Sciences, Chengdu 610066, China; Department of Biological Science, College of Life Sciences, Sichuan Normal University, Chengdu 610101, Sichuan, China; Plant Functional Genomics and Bioinformatics Research Center, Sichuan Normal University, Chengdu 610101, Sichuan, China; Department of Biological Science, College of Life Sciences, Sichuan Normal University, Chengdu 610101, Sichuan, China; Plant Functional Genomics and Bioinformatics Research Center, Sichuan Normal University, Chengdu 610101, Sichuan, China; Department of Biological Science, College of Life Sciences, Sichuan Normal University, Chengdu 610101, Sichuan, China; Plant Functional Genomics and Bioinformatics Research Center, Sichuan Normal University, Chengdu 610101, Sichuan, China

## Abstract

Polyploid plants typically display advantages on some agronomically important traits over their diploid counterparts. Extensive studies have shown genetic, transcriptomic, and epigenetic dynamics upon polyploidization in multiple plant species. However, few studies have unveiled those alternations imposed only by ploidy level, without any interference from heterozygosity. Cultivated potato is highly heterozygous. Thus, in this study, we developed two homozygous autotetraploid lines and one homozygous diploid line in parallel from a homozygous diploid potato. We confirmed their ploidy levels using chloroplast counting and karyotyping. Oligo-FISH and genome re-sequencing validated that these potato lines are nearly homozygous. We investigated variations in phenotypes, transcription, and histone modifications between two ploidies. Both autotetraploid lines produced larger but fewer tubers than the diploid line. Interestingly, each autotetraploid line displayed ploidy-related differential expression for various genes. We also discovered a genome-wide enrichment of H3K27ac in genic regions upon whole-genome doubling (WGD). However, such enrichment was not associated with the differential gene expression between two ploidies. The tetraploid lines may exhibit better resistance to cold-induced sweetening (CIS) than the diploid line in tubers, potentially regulated through the expression of CIS-related key genes, which seems to be associated with the levels of H3K4me3 in cold-stored tubers. These findings will help to understand the impacts of autotetraploidization on dynamics of phenotypes, transcription, and histone modifications, as well as on CIS-related genes in response to cold storage.

## Introduction

Polyploid plants typically display enlarged organ, altered architecture, enhanced growth vigor, and improved tolerance to abiotic stresses [1–3], as well as novel phenotypes compared to their diploid progenitors [4], which thus may not only provide them a fitness advantage for short-term adaptation [3], but also offer a potential breeding approach to increase yields in crops without gene manipulation [5–7]. Polyploids typically include autopolyploids and allopolyploids [3, 8]. Autopolyploids are considered to arise within a single species by the doubling of homologous genome, whereas allopolyploids arise through the merging of genomes from different species (hybridization) and subsequent doubling [3, 8].

A number of allopolyploid plant species, including important crops, have been well studied, such as bread wheat [9], *Brassica juncea* [10], strawberry [11], and cotton [12]. Investigations of allopolyploidization in such plant species mainly focus on genetic variation, changes in gene expression and DNA methylation [9, 10, 12, 13]. Since the merge of distinct subgenomes affecting allopolyploid offspring on both ploidy level and heterozygosity, it is challenging to discuss the effect of ploidy level on such changes alone without any interferences from heterozygosity. The example of maize suggests that gene expression is altered by both hybridization and ploidy change [14]. Therefore, whole-genome doubling (WGD) of homozygous diploid plants may provide an ideal system to assess the ploidy level on genetic, gene expression, and epigenetic variations. Compared to allopolyploidization, few studies have been dedicated to investigating the impacts of autopolyploidization on genetic and epigenetic changes [15–19]. In addition, most of these studies only focus on alternation of DNA methylation as the epigenetic impact caused by autopolyploidization, whereas evidence for changes in histone modifications are limited, such as active histone marks H3K4me3 [20] and H3K27ac [21], as well as repressive histone mark H3K27me3 [22]. Thus, the dynamics of histone modifications upon autopolyploidization remain elusive.

Potato is the most important non-grain food crop. After harvesting, potato tubers are usually stored at cold temperature to control diseases and prevent sprouting. However, along with the cold storage, starch is broken down into reducing sugars (glucose and fructose) in potato tubers. This process is called cold-induced sweetening (CIS) [23]. The accumulation of reducing sugars further results in an unsatisfactory dark color and accumulation of acrylamide in processing potato products with high temperature [24–26]. CIS is a long-lasting challenge to the potato industry. Successful attempts to improve the resistance to CIS are achieved mainly by silencing/mutating a key gene called *vacuolar invertase* (*VINV*) using RNAi [26], TALENs [27], and CRISPR-Cas9 [28]. However, the regulation of a number of other key genes involved in CIS is unclear [29]. Polyploid plants are often associated with better tolerance to abiotic stresses over their diploid progenitors [3], such as cold [30]. Thus, understanding the expression and epigenetic regulation of CIS-related genes at different ploidy levels may provide clues for improving resistance to CIS in potato.

Cultivated potato (*Solanum tuberosum*, 2n = 4x = 48) is highly heterozygous [31]. However, a doubled monoploid, DM1–3516 R44 (*S. tuberosum* group Phureja, 2n = 2x = 24, referred to as DM 1–3) with its homozygous and well-sequenced genome [32] has been a valuable source for genetic, cytogenetic, and epigenetic studies. DM 1–3 and its derived tetraploids provide an ideal system and allow us to study the impacts of ploidy levels on genetic and epigenetic variations on a genome-wide scale, with minimal interference from heterozygosity. In this study, we developed two tetraploid potato lines from DM 1–3 through WGD using callus culture. Both tetraploid derivatives displayed phenotypic polymorphisms, including larger tuber size, over the parallel diploid line, which failed in WGD. Chloroplast counting and karyotyping confirmed their ploidy levels. No structural variations were observed between the tetraploid and diploid lines at chromosome level using Oligo-FISH. Genome re-sequencing revealed limited DNA sequence variations among these lines, indicating these potato lines are nearly homozygous. Interestingly, each homozygous tetraploid line displayed ploidy-related differential expression for various genes. We discovered a genome-wide enrichment of H3K27ac in genic regions upon WGD. However, that enrichment was not associated with differential gene expression between two ploidies. The tetraploid lines may exhibit better resistance to CIS than the diploid line, indicated by lower levels of reducing sugars in cold tubers. The differences in reducing sugar content among three potato lines are coincident with the expression of key genes involving in CIS, which seems to be associated with the levels of H3K4me3. Our study will provide useful germplasm and help to understand the impacts of WGD on multiple aspects from phenotypes, chromosomes, to genetic and epigenetic variations, as well as on CIS-related genes in response to cold storage.

## Results

### Development of tetraploid potato lines from the homozygous diploid DM 1–3

The homozygous diploid potato DM 1–3 and its homozygous tetraploid derivatives would provide a great opportunity to investigate the impact of ploidy level on phenotypes, gene expression and histone modifications, with minimal interferences from heterozygosity. Therefore, we developed two tetraploid potato lines (DM4X-13 and DM4X-17) with doubled chromosome number from DM 1–3 using callus culture [33]. In addition, we selected one diploid line (DM2X-new) in parallel, which failed in chromosome doubling, to eliminate the influence from the hormone treatment during callus culture.

**Figure 1:**
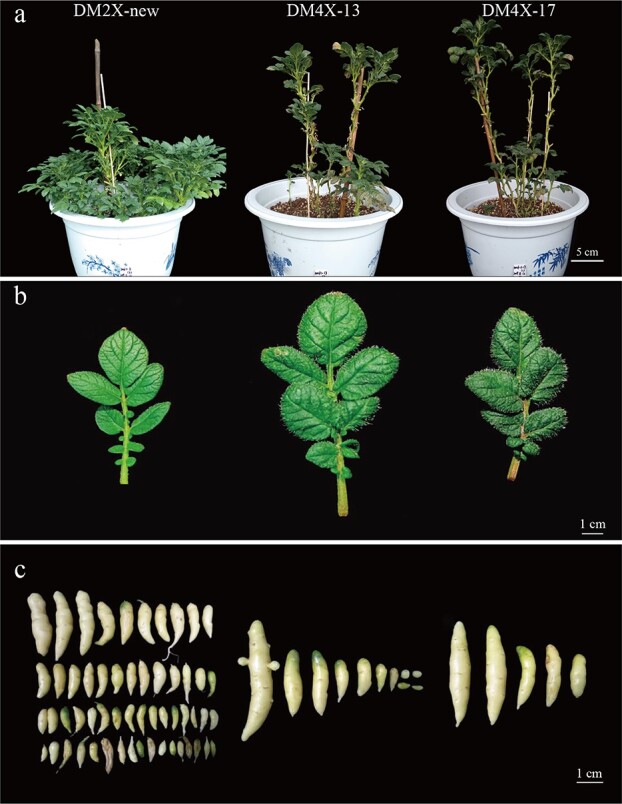
Phenotypes of the diploid and tetraploid potato lines. **a** Plant architecture. **b** Leaflets. **c** Tubers from single plant.

Typical variations in phenotypes from different ploidy levels were observed, including increased plant height, stem diameter, leaf size and upright plant architecture with less branch number in both DM4X-13 and DM4X-17, compared to those in DM2X-new (Fig. 1a and b; Table S1, see online supplementary material). Both tetraploid lines displayed larger anthers and styles than DM2X-new (Fig. S1, see online supplementary material). These morphological differences between the diploid and tetraploid potato lines are similar to other plant species, such as tomato [34], *Arabidopsis* [35], and watermelon [36].

Tetraploid potato tends to produce fewer but larger tubers than diploid ones [37, 38]. A similar trend was also observed between the tetraploid lines and DM2X-new (Fig. 1c; Table S1, see online supplementary material). Both DM4X-13 and DM4X-17 produced significantly fewer tubers (Fig. S2a, see online supplementary material) and lower total tuber yield than DM2X-new (*t* test, *P* < 0.01) (Fig. S2b, see online supplementary material). However, the average tuber weight of both tetraploid lines, particularly for DM4X-17, tends to be higher compared to that of DM2X-new (Fig. S2c, Table S1, see online supplementary material). Thus, the elevation of the ploidy level from diploid to tetraploid may be associated with an increase in the single tuber weight, rather than the total yield.

### Cytogenetic verification using chloroplast counting and oligo-FISH

Because the average ploidy level can be quickly estimated by using the chloroplast number in the guard cells of a stoma [39], we evaluated these newly developed potato lines and found that the chloroplast number of DM4X-13 and DM4X-17 ranged from 15–21 and from 16–26, respectively (Fig. S3, see online supplementary material), while the chloroplast number of DM2X-new varied from 8 to 14, which is similar to the diploid progenitor DM 1–3 with chloroplast number 7–14 (Fig. S3, see online supplementary material). This result indicates that the number of the chromosomes is likely doubled in both DM4X-13 and DM4X-17, while it remains at diploid level in DM2X-new.

**Figure 2:**
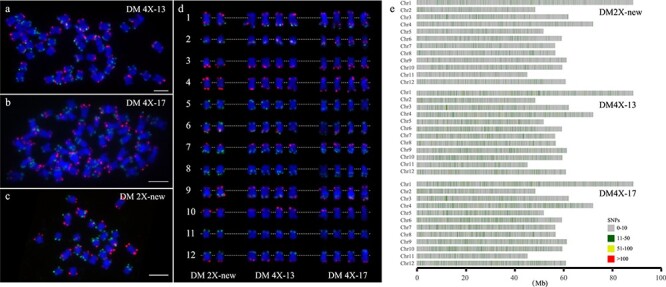
Oligo-FISH and genome re-sequencing for the diploid and autotetraploid lines. The DM4X-13 (**a**), DM4X-17 (**b**), and DM2X-new (**c**) panels show a complete metaphase cell from potato with two oligo-FISH probes of PB9446 (green) and PB8495 (red), respectively. Bars = 10 μm, *n* = 30. Homologous chromosomes in the middle panel (**d**) were digitally excised from the same cells and paired. The centromeres of the chromosomes are aligned by a dotted line. (**e**) The frequency of heterozygous SNPs in three potato lines. Windows with ≤10 heterozygous SNPs are filled with gray, window size = 10 kb.

We further confirmed the ploidy level for each line by karyotyping. Both DM4X-13 and DM4X-17 contain 48 chromosomes (Fig. 2a and b), while DM2X-new has 24 chromosomes (Fig. 2c). In addition, we employed Oligo-FISH with potato chromosome-specific probes [40] to evaluate structural variations at chromosome level between the diploid and tetraploid lines. The FISH results displayed no visible changes in signal localization among DM2X-new, DM4X-13, and DM4X-17 (Fig. 2d), suggesting both tetraploid lines contain the full set of the doubled genome and likely there is no large chromosomal structural variation among these lines.

### Sequence variation evaluated by genome re-sequencing

To further identify variation at DNA sequence level, genomes from the three newly developed lines were re-sequenced (Table S2, see online supplementary material). A total of 277 million (M) raw pair-end (PE) reads were obtained for these lines. Approximately, 90% of the raw reads were mapped to the genome assembly DM 1–3 (v4.04) [41], covering 20X for DM2X-new, 31X for DM4X-13 and 32X for DM4X-17, respectively. DM2X-new displayed 94.1% homozygosity, while DM4X-13 and DM4X-17 showed 89.8% and 89.3% homozygosity, respectively (Fig. 2e), suggesting these three potato lines are nearly entirely homozygous.

Compared to the reference genome of DM 1–3, we identified a limited number of sequence variants in DM2X-new, including 11 217 structural variations (SVs), 195 121 single nucleotide polymorphisms (SNPs) and 2128 insertion/deletions (Indels) (Fig. S4, centromere regions indicated [42], see online supplementary material). In the tetraploid lines, we detected a slightly larger number of sequence variants (Fig. S4, see online supplementary material). Noticeably, in DM2X-new, 6.0% (2358/39400) and 4.7% (1860/39400) of the potato protein-coding genes were associated with sequence variants in the putative promoters [1 kb upstream regions of the transcription start sites (TSSs)] and exons, respectively (Fig. S5a, see online supplementary material). Approximately 1.5% (588/39400) of genes had sequence variants in both regions in DM2X-new (Fig. S5a, see online supplementary material). In the tetraploid lines, slightly more genes were associated with sequence variants in the corresponding regions (Fig. S5a, see online supplementary material).

In addition, comparison between DM2X-new and the tetraploid lines revealed that only a small number of genes (2.2% of 39 400) harboring sequence variants in exons were unique to DM2X-new, while 4.2% (1650) and 4.1% (1605) of the genes with sequence variants in exons were specific to DM4X-13 and DM4X-17, respectively (Fig. S5b and c, see online supplementary material). Together, these results indicate that there are limited differences in DNA sequence between the newly developed diploid and tetraploid lines, particularly in the putative promoters and the coding regions.

### Impact of WGD on gene expression

**Figure 3:**
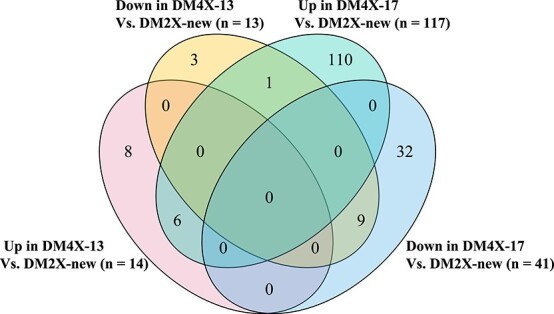
Venn diagram of DEGs between two independent WGD events. Each ellipse represents the number of the DEGs between a given tetraploid line and DM2X-new. Up represents up-regulated genes and down represents down-regulated genes. WGD events include comparison between DM2X-new and DM4X-13, and comparison between DM2X-new and DM4X-17.

To explore any variation in gene expression pattern between the diploid and tetraploid lines, we conducted RNA-seq for each line using room-temperature stored tubers (named RT tubers) (Table S3, see online supplementary material). Gene expression level between two biological replicates from each line was highly correlated (Pearson’s correlation *R^2^* = 0.96–0.98, *P* < 2.2e-16) (Fig. S6a, see online supplementary material). In comparison with DM2X-new, a total of 27 genes (14 up-regulated and 13 down-regulated) were statistically differentially expressed in DM4X-13, while 158 genes (117 up-regulated and 41 down-regulated) were differentially expressed in DM4X-17 (Fig. S6b, see online supplementary material). It is interesting that the differentially expressed genes (DEGs) from the two independent WGD events (DM2X-new vs. DM4X-13; DM2X-new vs. DM4X-17) overlapped only in a small proportion (six up-regulated genes and nine down-regulated genes), resulting in a number of DEGs that were unique to each WGD event (Fig. 3), leading us to suggest that ploidy elevation from diploid to tetraploid does not affect gene expression in tubers in consistent ways across the tetraploid lines. Comparison between the original line DM 1–3 and the tetraploid lines also revealed a considerable number of DEGs (14%–30%) were unique to each WGD event. Similar patterns are evidenced by WGD-wild potato species [2].

Interestingly, investigation of the six up-regulated genes in both WGD events (Fig. 3) revealed that four genes have been reported to be involved in abiotic stresses [43–45]. In addition, gene ontology (GO) analysis for 110 genes, which were specifically up-regulated in DM4X-17 in the two WGD events (Fig. 3), showed that most of them were enriched in functions related to responses to stresses and stimulus (Fig. S7, see online supplementary material). Similar up-regulation of gene expression related to enhanced stress tolerance was found in autotetraploid Mulberry [19] and autotetraploid Birch [46]. Polyploid plants are thought to be associated with improved ability to tolerate harsh environments [47]. Thus, these results may indicate that elevation of ploidy from diploid to tetraploid enhances the ability for potato to tolerate multiple stresses, especially for DM4X-17.

### WGD induced the enrichment of active histone modification H3K27ac

**Figure 4:**
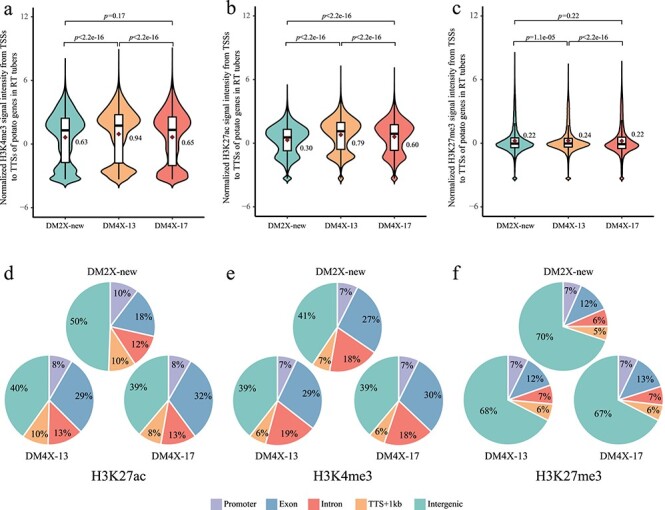
Profiles of histone modifications in RT tubers. H3K4me3 (**a**), H3K27ac (**b**), and H3K27me3 (**c**) levels in the gene body regions of the potato genes in DM2X-new, DM4X-13, and DM4X-17. Signal was quantified from TSS to transcription terminate site (TTS) for each gene and showed with log2 transformation. Statistical significance was tested using Wilcoxon signed-rank test. Median value was indicated in each panel. Genomic distribution of histone modification peaks identified in RT tubers for H3K27ac (**d**), H3K4me3 (**e**), and H3K27me3 (**f**). Peak was assigned to a genomic feature if it is occupied by the center of the peak. Promoter indicates the region 1 kb upstream from TSS. TTS + 1 k indicates the region 1 kb downstream from TTS. Intergenic refers to the region 1 kb away from any TSS or TTS.

We conducted chromatin immunoprecipitation followed by sequencing (ChIP-seq) for each potato line, with antibodies against active histone marks of H3K4me3 and H3K27ac, as well as a repressive histone mark H3K27me3 (Table S4, see online supplementary material). Comparison of the histone modification levels in genic regions of all potato genes revealed that DM4X-13 displayed generally higher levels of H3K4me3 than DM2X-new (Fig. 4a). Both tetraploid lines showed higher levels of H3K27ac than DM2X-new (Fig. 4b), while less difference in the levels of H3K27me3 was observed (Fig. 4c), indicating the tetraploid lines seem to be enriched with the active histone modifications in genic regions. In addition, the diploid progenitor DM 1–3 was associated with the lowest levels of H3K4me3 among all tested lines (Fig. S8a, see online supplementary material), while the levels of H3K27ac in DM 1–3 fell between DM2X-new and the tetraploid lines (Fig. S8b, see online supplementary material). Thus, it is indicated that tetraploidization may induce the enrichment of H3K27ac, regardless of the hormone treatment during tissue culture.

We identified the enriched regions (peaks) of H3K4me3, H3K27ac, and H3K27me3 for all lines, respectively (Fig. S9a–c, centromere regions indicated [42], see online supplementary material). The peaks of the three histone marks were localized in the euchromatin regions of each chromosome in all potato lines (Fig. S9d–f, see online supplementary material). It is interesting that a marked difference in genomic distribution of H3K27ac peaks was found in exons and intergenic regions between two ploidies (*z* test, *P* < 0.01) (Fig. 4d). Approximately, two-thirds more H3K27ac peaks from the tetraploid lines (29% for DM4X-13 and 32% for DM4X-17) were observed in exons comparing to that from DM2X-new (18%) (Fig. 4d). In contrast, the distribution of both H3K4me3 and H3K27me3 peaks was generally similar between DM2X-new and the tetraploid lines (Fig. 4e and f).

We further observed a considerable amount of H3K27ac peaks specific to DM4X-13 (53.7%, 27 759) and DM4X-17 (35.3%, 8097) in two WGD events (Fig. S10a, see online supplementary material). These specific peaks as well as their cognate genes overlapped only in a small proportion between the two tetraploid lines (21.3% of peaks and 26.5% of genes relative to DM4X-13). A similar trend was also found for H3K27ac peaks located in exons (Fig. S10b, see online supplementary material). In contrast, only a small proportion of H3K4me3 and H3K27me3 peaks were found to be specific to the tetraploid lines (Fig. S10c and d, see online supplementary material). Thus, it is indicated that WGD triggers the emergence of a considerable amount of H3K27ac in genic regions, but a limited number of H3K4me3 and H3K27me3 enriched regions. Unexpectedly, in comparison to DM2X-new, 99.9% (12 406/12420) and 99.5% (4189/4209) of genes associated with tetraploid-specific H3K27ac peaks were either silenced or constitutively expressed in DM4X-13 and DM4X-17, respectively. Collectively, these results suggest that WGD, at least for potato, may induce the enrichment of the active histone modification H3K27ac, which seems to have limited effect on alternation of transcription.

### WGD-induced differential expression tends to be independent to sequence variations

**Figure 5:**
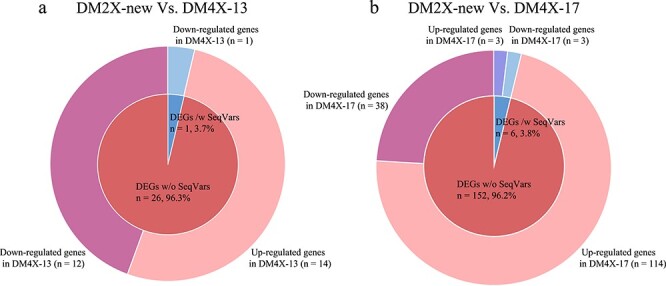
Ploidy-related DEGs associated with sequence variants. Double pie chart shows the number and ratio of DEGs between DM2X-new and DM4X-13 (**a**) as well as between DM2X-new and DM4X-17 (**b**), with and without sequence variants. SeqVars represents sequence variants, including SNPs, InDels, and SVs. Genes were associated with sequence variants, if any type of variation was observed in their body regions and 1 kb upstream regions from TSSs.

To interrogate whether differential gene expression between ploidies is potentially associated with variation in DNA sequence, we identified sequence variants (SVs, indels and SNPs) in exon regions as well as putative promoters of the DEGs from each WGD event. In comparison with DM2X-new, we found sequence variants in only a single (3.7% of 27) DEG in DM4X-13, while the remaining 26 DEGs (96.3%) were not associated with any sequence variants in the corresponding regions (Fig. 5a). Similarly, we found six (3.8% of 158) DEGs were associated with sequence variants in DM4X-17 compared to DM2X-new, while the sequence from both regions of 152 (96.2% of 158) DEGs in DM4X-17 were identical to that in DM2X-new (Fig. 5b). This result indicates that variation in DNA sequence does not tend play a major role in the regulation of the differential gene expression between the homozygous diploid and tetraploid lines. Further observation on histone modifications for these DEGs without any sequence variants revealed that the alternation of the expression between two ploidies tends to be independent to the enrichment of the histone modifications (Fig. S11, see online supplementary material), indicating these DEGs might be regulated by other epigenetic modifications rather than the tested ones.

### Dynamics of gene expression in response to cold storage

**Figure 6:**
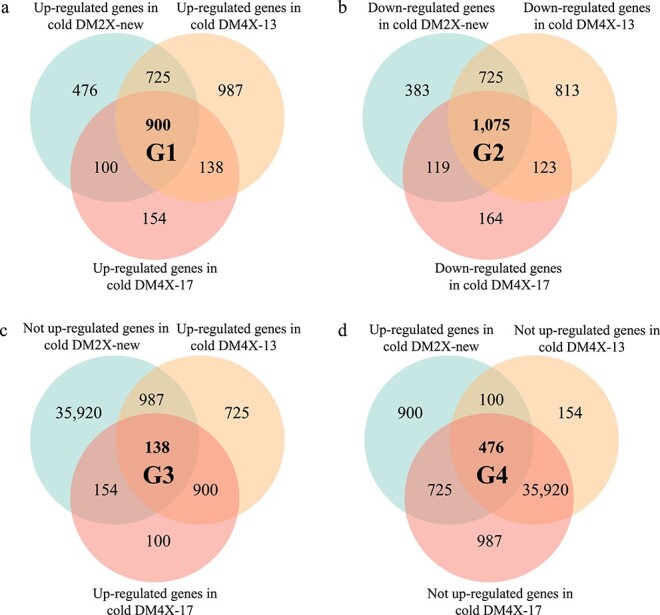
Cold-induced DEGs behaved in three potato lines. For each group, DEGs were first called between RT and cold tubers for each line. Among the three lines, cold-responsive DEGs either behaved consistently (G1 and G2) or differently (G3 and G4). The cold-responsive genes were up-regulated in three lines (**a**), down-regulated in three lines (**b**), only up-regulated in both autotetraploid lines (**c**), and only up-regulated in the diploid line (**d**). ‘Not up-regulated genes’ represent genes that are down-regulated, constitutively expressed and silenced in other line(s) in response to cold storage.

To investigate any differences in gene expression pattern in response to cold storage of tubers between the diploid and tetraploid lines, we developed additional transcriptome data from tubers stored under cold temperature (4°C) for each line (named cold tubers) (Table S3, see online supplementary material). The overall gene expression in cold tubers between two biological replicates of each line was highly correlated (Pearson’s correlation *r* = 0.81–0.93, *P* < 2.2e-16) (Fig. S6a, see online supplementary material). In DM2X-new, we found that a total of 2201 and 2302 genes were statistically significantly up- and down-regulated upon cold storage, respectively (Fig. S12, see online supplementary material). A slightly larger number of DEGs (2750 up-regulated genes and 2736 down-regulated genes) was observed in DM4X-13 upon cold storage, while only 1292 up-regulated genes and 1481 down-regulated genes were identified in DM4X-17 (Fig. S12, see online supplementary material). We categorized these DEGs into four groups based on their expression behavior upon cold storage, including genes: G1, up-regulated in three lines (*n* = 900) (Fig. 6a); G2, down-regulated in three lines (*n* = 1075) (Fig. 6b); G3, up-regulated in the tetraploid lines, but not up-regulated (down-regulated, constitutively expressed and silenced) in the diploid line (*n* = 138) (Fig. 6c); and G4, up-regulated in the diploid line, but not up-regulated in the tetraploid lines (*n* = 476) (Fig. 6d).

We found a large proportion of up- (G1) and down-regulated genes (G2) behaved consistently in the three lines upon cold storage (Fig. 6a and b), which was substantially more than DEGs from the other two groups (Fig. 6c and d), suggesting the direction of the differential gene expression in response to cold storage tends to be similar among the three lines regardless of the ploidy level. The GO terms for both G1 and G2 were mainly related to organelles and ribonucleoprotein complex (Fig. S13a and b, see online supplementary material), indicating that cold storage appears to affect the function of the basic cell components. Noticeably, DM4X-13 showed the largest changes in gene expression in both G1 and G2 upon cold storage than the other two lines (Fig. S14a and b, see online supplementary material), whereas DM4X-17 displayed the least variation in gene expression (Fig. S14a and b, see online supplementary material). The difference in the degree of the differential expression among the three lines was also supported by individual cold-responsive genes [48–54] (Fig. S14d, see online supplementary material).

For G3, these genes displayed larger changes in expression in DM4X-13 than in DM4X-17 in response to cold storage (Fig. S14c, see online supplementary material). They were found to be enriched in functions related to response to stress and metabolic processes (Fig. S13c, see online supplementary material). Two heat-shock protein genes (PGSC0003DMG400003122 and PGSC0003DMG400028744) from G3, whose closest homologs participate in responding heat stress in peppers [50], tomatoes [51], potato [51, 52], and cold stress in soybeans [53], were also detected to be significantly up-regulated in the tetraploid lines (Fig. S14e, see online supplementary material). Therefore, it is indicated that the tetraploid lines might be associated with better tolerance to cold stress to some degree.

**Figure 7:**
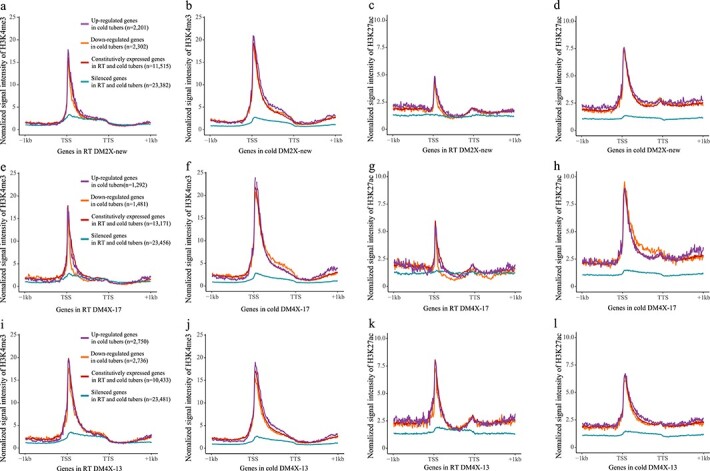
Profiles of histone modifications associated with potato genes in response to cold storage. The levels of H3K4me3 (**a**, **b**) and H3K27ac (**c**, **d**) associated with potato genes in RT tubers (**a**, **c**) and cold tubers (**b**, **d**) of DM2X-new. The levels of H3K4me3 (**e**, **f**) and H3K27ac (**g**, **h**) associated with potato genes in RT tubers (**e**, **g**) and cold tubers (**f**, **h**) of DM4X-17. The levels of H3K4me3 (**i**, **j**) and H3K27ac (**k**, **l**) associated with potato genes in RT tubers (**i**, **k**) and cold tubers (**j**, **l**) of DM4X-13. Genes were divided into 100 bins and aligned together from TSSs to TTSs. Genes flanking regions (±1 kb) were analysed in 100 bins. Histone modification signal was measured by quantifying the mid-point of ChIP-seq PE reads within each bin and normalizing by ChIP-seq read number per bp genome region per million mapped reads.

In G4, 476 DEGs were found to be only up-regulated in DM2X-new compared to the tetraploid lines upon cold storage (Fig. 7d). Over 98% of them were either constitutively expressed or silenced in both tetraploid lines. The GO analysis revealed that these DEGs were mainly related to thylakoids (Fig. S13d, see online supplementary material). Reduction in efficiency of photosynthesis can be frequently observed in multiple plant species as a mean of protection for the photosynthetic apparatus in response to environmental stress, such as cold stress for *Vitis vinifera* [55], *Cistus albidus* and *Quercus ilex* [56]. However, the potato tuber is not a primarily photosynthetic organ [57]. The relatively stable gene expression in the tetraploid lines might be associated with less greening in tuber skin.

### Dynamics of histone modifications in response to cold storage

We conducted additional ChIP-seq with antibodies against H3K4me3, H3K27ac, and H3K27me3, separately, using the cold-stored tubers from each line (Table S4, see online supplementary material). Upon cold storage, both DM2X-new and DM4X-17 displayed overall increased levels of active histone modifications (H3K4me3 and H3K27ac) around TSSs of the DEGs and constitutively expressed genes (Fig. 7a–h). In contrast, DM4X-13 showed generally reduced levels of both active histone modifications around TSSs of these genes (Fig. 7i–l). For the repressive mark H3K27me3, all lines displayed either reduced or similar levels in the corresponding regions (Fig. S15, see online supplementary material).

We identified 57 654, 54 249, and 53 462 H3K4me3 peaks in cold tubers from DM2X-new2 DM4X-13, and DM4X-17, respectively (Fig. S9a–c, see online supplementary material). Approximately, 12.4% (7121/57654), 11.26% (6106/54249), 19.56% (10 457/53462) peaks were specific to cold tubers from DM2X-new, DM4X-13, and DM4X-17, respectively (Fig. S16a, see online supplementary material). In contrast to H3K4me3, a large number of cold-specific peaks were discovered for H3K27ac (39%–72%) (Fig. S16b, see online supplementary material) as well as for H3K27me3 (46%–59%) (Fig. S16c, see online supplementary material) in each line. Thus, cold stress induces a considerable number of H3K27ac and H3K27me3 enriched regions, but a limited number of H3K4me3 enriched regions, indicating H3K27ac and H3K27me3 may play a regulatory role in response to cold storage. Furthermore, comparing to cold-specific H3K4me3 peaks from DM2X-new, a number of cold-specific H3K4me3 peaks were only detected in tetraploid lines (*n* = 3990 and *n* = 7181), and a large proportion of these cold-specific peaks were unique to each tetraploid line (*n* = 2579 and *n* = 6407) (Fig. S16a, see online supplementary material). Similar patterns were observed for H3K27ac (Fig. S16b, see online supplementary material) and H3K27me3 (Fig. S16c, see online supplementary material), suggesting that the tetraploid lines were associated with diverse histone modifications in response to cold storage.

We further analysed the changes of histone modifications for genes from G1–G4 upon cold storage and found that each group displayed similar patterns with the least variations of H3K4me3 (Fig. S17a–d, see online supplementary material) and H3K27ac (Fig. S17e–h, see online supplementary material) in DM4X-13, but a relatively similar degree of variation between DM2X-new and DM4X-17 (Fig. S17a–h, see online supplementary material). In each gene group, DM4X-17 displayed generally larger variation in H3K27me3 than the other two lines upon cold storage (Fig. S17i–l, see online supplementary material). The cold-induced variation in histone modifications was not associated with the changes of the gene expression, suggesting that other epigenetic modifications may potentially play a major role in the regulation of differential expression in response to cold.

### Resistance of cold-induced sweetening in the tetraploid lines

**Figure 8:**
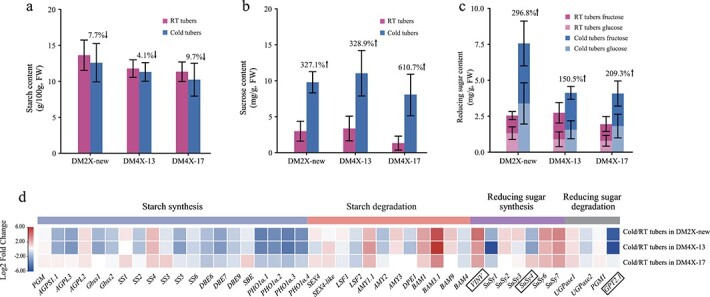
Comparison of starch and sugar contents and the expression of CIS-related genes among three potato lines in response to cold storage. The content of starch (**a**), sucrose (**b**), and reducing sugar (**c**) in RT tubers and cold tubers from DM2X-new, DM4X-13, and DM4X-17. FW indicates fresh weight. The percentage above columns represents the change of content in cold tubers compared to RT tubers. Up-arrow indicates increase in cold tubers vs. RT tubers and down-arrow indicates decrease in cold tubers vs. RT tubers. Three biological replicates were used for the evaluation of starch and sugar contents. **d** Heatmap of fold change in expression upon cold storage in each line. The full names and expression levels are listed in Table S5.

To evaluate the degree of CIS in the newly developed potato lines, we measured the contents of starch, sucrose, and the reducing sugar (glucose and fructose) in both RT and cold tubers using high-performance liquid chromatography (HPLC). Upon cold storage, the starch content was reduced in both diploid and tetraploid lines (Fig. 8a), while the sucrose and the reducing sugar were increased (Fig. 8b and c). Coincident with cold-induced degradation of starch, most of the starch synthesis-related genes were down-regulated upon cold storage (Fig. 8d), while the starch degradation-related genes were generally up-regulated (Fig. 8d). In addition, the genes related to reducing sugar synthesis were overall up-regulated (Fig. 8d). For reducing sugar degradation, *Glucose 6-Phosphate/phosphate Transporter* (*GPT*2.1, PGSC0003DMG400005269), a highly tuber-specific expressed gene involved in transporting Glucose 6-Phosphate (G6P) [29], was substantially down-regulated in DM2X-new and DM4X-13, but remained similarly expressed in DM4X-17, detected by both RNA-seq (Fig. 8d) and quantitative real-time PCR (qRT-PCR) (Fig. S18, see online supplementary material). Collectively, these results indicate that cold storage triggers CIS in both diploid and tetraploid lines possibly through the differential expression of the key genes involved in the carbohydrate metabolism pathway.

Although both diploid and tetraploid lines contained similar level of starch in both RT and cold tubers (Fig. 8a), the accumulation of the sucrose and reducing sugar was obviously different upon cold storage (Fig. 8b and c). In cold tubers, DM4X-13 contained the highest amount of sucrose, while DM 4X-17 had the lowest amount of sucrose (Fig. 8b). The *Sucrose Synthesis 4* (*SuSy4*) (PGSC0003DMG400002895), a major tuber-specific *SuSy* gene [58, 59], encode proteins that catalyze the breakdown of the sucrose in tubers [60]. The sucrose contents of these lines are coincident with the expression levels of the *SuSy4* in cold tubers, with transcripts per kilobase million (TPM) = 345, 201, and 3472 in DM2X-new, DM4X-13, and DM4X-17, respectively (Fig. 8d;Fig. S18, see online supplementary material). Both tetraploid lines accumulated less reducing sugar than DM2X-new in cold tubers (*t* test *P* < 0.05) (Fig. 8c). In addition to *SuSy4*, the degradation of sucrose into glucose and fructose is also mediated by vacuolar acid invertase (VINV), a key enzyme involved in CIS [61]. Coincident with the accumulation of the reducing sugar in cold tubers of each line, the expression of *VINV* (PGSC0003DMG400013856) showed the highest level (TPM = 266) in DM2X-new compared to that in the tetraploid lines (TPM = 178 in DM4X-13 and TPM = 73 in DM4X-17) (Fig. 8d; Fig. S18, see online supplementary material). Collectively, the reduction in accumulation of the reducing sugar in tetraploid lines implies that these homozygous tetraploid lines might be associated with better resistance to CIS than the diploid line through the differential expression of the key genes in the starch metabolism pathway.

We further examined if the differential expression of the tested carbohydrate metabolism-related genes is potentially associated with sequence variation. None of these genes were associated with any types of sequence variants in their putative promoters and genic regions, except for the putative promoters of the *Starch Synthesis I* (*SS1*) and the *Debranching Enzyme 9* (*DBE9*). Therefore, the difference in the cold-induced differential expression of these genes among the three potato lines is likely independent to the genetic variation observed here. Investigation of histone modifications for the key genes *SuSy4* and *VINV* revealed their expression levels were associated with the levels of H3K4me3. In cold tubers, the H3K4me3 levels of *SuSy4* in DM2X-new (3.97), DM4X-13 (2.8), and DM4X-17 (4.45) were coincident with the expression levels of the gene in each line (Fig. S19, see online supplementary material). Similarly, *VINV*, which displayed the highest levels of H3K4me3 in cold tubers of DM2X-new, was expressed at the highest level than the tetraploid lines (Fig. S19, see online supplementary material). Collectively, we speculate that the homozygous tetraploid lines may display better resistance to CIS via epigenetic-mediated ectopic expression of some key CIS genes upon WGD.

## Discussion

### Phenotypic variations upon WGD

WGD in both autopolyploids and allopolyploids can lead to immediate phenotypic, genetic, and epigenetic changes and such polyploids might have adaptive advantage compared to their diploid progenitors [3]. In this study, both autotetraploid potato lines were associated with various phenotypic changes compared to their parallel diploid line (Fig. 1; Table S1, see online supplementary material), which are typical differences commonly found between diploid and polyploid from other plant species [34–36]. General observations show that tetraploid potato cultivars are associated with higher yield than diploid ones [7]. However, the total tuber yield of two autotetraploid lines in this study was significantly lower than that of the diploid line (Fig. S2b, Table S1, see online supplementary material), although both autotetraploid lines produced larger but fewer tubers (Fig. 1c; Table S1, see online supplementary material). Thus, the elevation of the ploidy level from homozygous diploid to tetraploid might contribute to the average tuber size rather than the total yield. A recent pioneer study in hybrid potato showed that F1 hybrids produced significant higher single plant tuber yield than their homozygous diploid parents [62], indicating the heterosis may impact the tuber yield. Thus, heterozygous diploid potato may produce comparable tuber yield to the tetraploid one. Deciphering the regulation of key genes between ploidies as well as between diverged subgenomes may provide clues for breeding targets.

### WGD impacts epigenetic modifications in the autotetraploid lines

The autotetraploid lines did exhibit sequence differences compared to the diploid line upon WGD (Fig. S4, see online supplementary material). Thus, the autotetraploid lines harbor more genetic diversity over the diploid line. Interestingly, 96% of the DEGs between two ploidies lacked any sequence polymorphisms in their putative promoters and coding regions (Fig. 5), suggesting epigenetic modifications may play a role in regulation of differential expression. However, the variations of all tested histone modifications appear to be not associated with the direction of the differential expression (Fig. S11, see online supplementary material), indicating the DEGs are likely to be regulated by other epigenetic modifications. DNA methylation has been discovered to play a regulatory role in differential expression between ploidies in multiple plant species, such as autotetraploid apple [63], *Aegilops tauschii* [64], *Artemisia annua* [65], *Arabidopsis thaliana* [66], and rice [17], implying that it is possible that the differential expression between the autotetraploid and diploid potato lines might be potentially mediated by differential DNA methylation.

Both autotetraploid lines displayed similar enrichment of H3K27ac in genic regions upon WGD (Fig. 4b). Histone modification H3K27ac is associated with transcription activation in plants [21]. However, both up- and down-regulated genes showed increased levels of H3K27ac upon WGD (Fig. S11a, see online supplementary material), suggesting the independence of H3K27ac to the alternation of transcription. Histone acetylation generally enhances chromatin accessibility by neutralization of the basic charge in histones [67, 68]. In plant, H3K27ac is enriched and peaked around TSSs, where chromatin is more accessible [69, 70]. We speculate that WGD-induced enrichment of H3K27ac might provide more accessible chromatin environment facilitating the access of transcription factors (TFs).

### WGD impacts gene expression unpredictably between each tetraploid line

An interesting question raised by this study is why WGD-induced phenotypically similar autotetraploid individuals were associated with divergent gene expression. Noticeably, the ploidy-related DEGs in DM4X-17 (*n* = 158) are close to six times more than the DEGs in DM4X-13 (*n* = 27) (Fig. S6b, see online supplementary material), and these DEGs overlapped in a very small proportion between the tetraploid lines (Fig. 3). Thus, WGD may affect expression of different genes in each autotetraploid lineage. Alteration of gene expression upon polyploidization has been well studied in both animal and plant systems and the alternation tends to be species-specific [3]. For instance, in *Arabidopsis*, two accessions (C24 and Ws) exhibited opposite direction of the expression change upon autotetraploidization [15]. Intriguingly, four autotetraploid individuals derived from two wild diploid potato species, respectively, displayed line-specific expression patterns with a large proportion of DEGs specific to each autotetraploid line [2], which is similar to our findings in the homozygous potato lines. Coincidently, the GO terms of the ploidy-related DEGs from the wild potato study as well as from our current study were mainly enriched in functions related to responses to stresses and cell components. None of these terms were associated with plant architecture, development, or growth. One possible explanation for the discrepancy between similar phenotypes and diverse gene expression between autotetraploid individuals would be that key genes related to core functions for development remain genetically and epigenetically identical between the derived autotetraploid individuals.

### Autotetraploid lines may be associated with better resistance to CIS

WGD-induced polyploids were associated with enhanced tolerance to abiotic stresses, such as cold [30], drought [16], and salt [17]. Similarly, the autotetraploid potato lines in this study may be associated with better tolerance to cold stress. We found that a number of genes involved in the carbohydrate metabolism pathway displayed diverse pattern of differential expression among the three potato lines in response to cold stress (Fig. 8d). The differences in differential expression pattern among the three lines are coincident with their amount of sucrose and reducing sugar contents (Fig. 8b and c). It is noted that cold tubers from both autotetraploid lines contained lower levels of reducing sugars than cold tubers from the diploid line, while the elevation of reducing sugars upon cold storage was less in the autotetraploid lines (Fig. 8c), implying that the autotetraploid lines may be associated with better tolerance to CIS. The differences in sucrose and reducing sugars contents among the three potato lines seem to be coincident with their differences in differential expression of key genes for sucrose degradation and reducing sugar synthesis, such as *SuSy4* and *VINV* (Fig. 8d; Fig. S18, see online supplementary material). In addition, their expression tended to be associated with the level of histone modification H3K4me3. Thus, understanding the epigenetic regulation of such key genes involved in CIS between ploidies may provide clues for the improvement of resistance to CIS.

## Materials and methods

### Plant materials

Tetraploid potato lines were developed from the homozygous diploid potato DM 1–3516 R44 (*S. tuberosum* Group phureja, 2n = 2x = 24) using callus culture [33] with hormones of NAA, BA, and GA_3_. The newly developed tetraploid lines and their diploid counterpart were subject to phenotyping, cytogenetic validation, genome re-sequencing, RNA-seq, and ChIP-seq. All potato lines were grown in a greenhouse under the photoperiod of 16 h/24°C daylight and 8 h/18°C darkness. Tubers were harvested 80 days after planting in the soil and were grouped for treatments at room temperature (22°C) and cold temperature (4°C) for 14 days, respectively. Upon temperature treatment, tubers were ground into fine powder under liquid nitrogen and stored at −80°C for experiments.

### Phenotyping

Plant architecture, plant height, stem diameter, branch number, leaf area, and flower were evaluated for all potato lines 60 days after planting. Tuber yield, number, and size were measured at harvest (80 days after planting).

### Chloroplast counting

The number of chloroplasts was scored in two guard cells of a stomata. Eight stomata were examined for each plant. Briefly, epidermal peels were taken from the abaxial side of leaves from 60-day old plants and immediately put on a slide with 1% iodine solution for staining. Cover slip was mounted after two minutes staining. Chloroplasts were then counted using a light microscope at 100 and 400$\times$ magnification.

### Chromosome counting and oligo-FISH

Root tips were cut from young potato plants and immediately treated with nitrous oxide under a pressure of 160 psi for 1 h for chromosome condensation. Fixation for root tips was performed immediately in the ethanol/acetic acid (3:1) solution, followed by a thorough wash three times using autoclaved distilled water. Root tips were then digested with 2% cellulase (Yakult Pharmaceutical, Tokyo, Japan) and 1% pectinase (Sigma, St. Louis, MO, USA) at 37°C for 90 minutes. The digested root tips were applied for chromosome preparation following the dropping method [71]. The number of chromosomes was counted in 30 individual cells from at least five plants for each line. The potato specific Oligo-FISH probes [40] were labeled using digoxigenin [72] and the hybridization was performed following the standard protocol [73]. Signal detection was conducted using rhodamine anti-digoxigenin with the DAPI antifade solution (Vector Laboratories, Newark, CA, USA). FISH images were captured using a fluorescence microscope (Zeiss Axio Scope A1) with a CCD camera (ORCA Flash4.0).

### Starch and sugar contents analysis

Both RT and cold tubers from each potato line were analysed for the starch, sucrose, glucose, and fructose contents. Starch was quantified based on the GB5009.9–2016 method. Sugar (sucrose, glucose, and fructose) contents were measured using HPLC [74], with the refractive index detector (RID) at 40°C, an amino column at 35°C, 10 μL injection volume, and 1 mL/min flow rate.

### RNA-seq and ChIP-seq

Tuber samples, which were used for starch and sugar contents analysis, were used for both RNA-seq and ChIP-seq. Tubers harvested from six individual plants were pooled as a biological replicate for each line. Two biological replicates of RNA-seq libraries were constructed for each temperature treatment and sequenced using an Illumina NovaSeq 6000 platform with 150 bp PE mode. All Illumina sequencing in this study was conducted using the same platform and mode in Novagene.

ChIP experiments were conducted following the published protocol [75], with antibodies against H3K4me3 (Abcam 8580), H3K27me3 (Millipore 07–449) and H3K27ac (Millipore 07–360), respectively. To achieve the highest resolution, chromatin was digested into monomer nucleosome pattern (~150 bp fragments) using MNase (Sigma N3755). Antibody-captured chromatin complex was precipitated using rProtein A sepharose beads (GE 17–1279-01). ChIP-DNA was separated from the precipitated chromatin for ChIP-seq library construction and sequencing.

### Reverse transcription and qRT-PCR

cDNA was synthesized using All-in-One First-Strand cDNA Synthesis SuperMix (EasyScript, #AE341–02) with oligo(dT)_20_. qRT-PCR for each gene was performed with SYBR Green I T5 Fast qPCR Mix (TsingKe, #TSE202), using a RT-PCR cycler (CFX Connect Bio-Rad). The qRT-PCR parameters were set with initial denaturation of 94°C for 30 s, and 45 cycles of 94°C for 5 s, 54°C for 15 s, and final extension of 72°C for 10 s. Three technical replicates and two biological replicates from each treatment were used for quantifying the expression. The reference gene *EF1α* was employed to normalize the expression of each gene, using the calculation of 2^-ΔΔCt^. The gene-specific primers used for each gene are listed in Table S6 (see online supplementary material).

### RNA-seq and ChIP-seq data analysis

Raw reads generated from RNA-seq and ChIP-seq were first processed for quality control using fastp program (https://github.com/OpenGene/fastp) [76] with default parameters. Cleaned RNA-seq reads were mapped to the DM 1–3 potato genome assembly (PGSC v4.04 [41]), using Hisat2 (v2.0.4) [77]. The expression values (TPM) of the annotated potato genes were evaluated using Stringtie (v2.1.5) [78]. The DEGs were identified and used for downstream analyses if they were detected by both DEseq2 (v1.32.0) [79] and Cuffdiff (v2.21) [80] with FDR <0.01. The programs for data processing and statistical tests were written and conducted in either Perl or R (https://www. r-project.org).

Similarly, cleaned ChIP-seq reads were mapped to the same genome assembly [41] using BWA ‘mem’ algorithm [81]. Reads were retained for further analyses if they were mapped to a unique position in the genome. The histone modification signal was defined as the mid-point of the uniquely mapped PE reads. The level of a histone modification within an interval was quantified by calculating histone modification signals and normalizing to length of the interval, PE read number per million uniquely mapped reads, as well as IgG. Histone modification enriched regions were identified using MACS2 sofware [82].

### Gene ontology enrichment

Potato genes were used to find homologous sequences with the highest similarity in *Arabidopsis*, using Blastp program (BLAST 2.9.0). The enriched GO terms were screened with the program of agriGO v2.0 [83], using *Arabidopsis* homologous protein sequences. Fisher’s exact test was applied for the GO enrichment test with Benjamini-Hochberg FDR *P*-value normalization. GO terms from all annotated *Arabidopsis* genes were set as background for each enrichment test.

### Variant calling

Genomic DNA isolated from young leaves was subject to library construction and sequencing. The raw reads were first cleaned using fastp program with default parameters, followed by the alignment to the PGSC v4.04 [41] using the BWA ‘mem’ [81] with default parameters. The mapped reads were further filtered using Samtools [84] with Q20 followed by ‘markdup’ [84] with default parameter for PCR duplication removal. The GATK program (https://gatk.broadinstitute.org) was used to detect SNPs and InDels. SNPs were hard-filtered with QD less than 2.0, MQ less than 40.0, FS greater than 60.0, SOR greater than 3.0, MQRankSum less than −12.5, ReadPosRankSum less than −8.0. InDels were filtered with QD less than 2.0, FS greater than 200.0, SOR greater than 10.0, MQRankSum less than −12.5, ReadPosRankSum less than −20.0. InDels included insertion or deletion with length of 1 to 10 bp. Structural variations were detected using Delly software [85] with default parameters. Structural variations included insertion, deletion, inter/intra chromosomal translocation and inversion.

## Data visualization

All data were visualized using R program (https://www.r-project.org). Circos plots were drawn using OmicCircos package (https://bioconductor.org/packages/OmicCircos/).

## Acknowledgements

This work was supported by grants from the National Natural Science Foundation of China (31900386 to Z.Z.), Sichuan Science and Technology Program (2021YFH0025 to Z.Z. and 2021YFYZ0019 to B.Z. and Z.Z.), State Key Laboratory of Crop Gene Exploration and Utilization in Southwest China at Sichuan Agricultural University (SKL-KF202205 to B.Z.), State Key Laboratory of Crop Biology Open Fund (2020KF01 to B.Z.). All data were visualized using R program (https://www.r-project.org). Circos plots were drawn using OmicCircos package (https://bioconductor.org/packages/OmicCircos/).

## Author contributions

Z.Z. and B.Z. designed and supervised the study. Z.Z. and B.Z. developed the plant materials. H.G. and M.Z. performed the data analysis. G.Z., L.H., C.Y., M.W. and D.Z. performed the experiments. H.G., M.Z., G.Z., J.H., W.H. and Z.Z. prepared the manuscript. All of the authors read and approved the manuscript.

## Data availability

Raw reads generated from potato in this study are available from National Center for Biotechnology Information (NCBI) under project number PRJNA873910.

## Conflict of interest statement:

The authors declare that they have no competing interests.

## Supplementary data

Supplementary data is available at *Horticulture Research* online.

## Supplementary Material

Web_Material_uhad017

## References

[1] Corneillie S , De Storme N, Van Acker R et al. Polyploidy affects plant growth and alters cell wall composition. Plant Physiol. 2019;179:74–87. 30301776 10.1104/pp.18.00967 PMC6324247

[2] Fasano C , Diretto G, Aversano R et al. Transcriptome and metabolome of synthetic solanum autotetraploids reveal key genomic stress events following polyploidization. New Phytol. 2016;210:1382–94. 26915816 10.1111/nph.13878

[3] Van de Peer Y , Mizrachi E, Marchal K. The evolutionary significance of polyploidy. Nat Rev Genet. 2017;18:411–24. 28502977 10.1038/nrg.2017.26

[4] Fawcett JA , Maere S, Van De Peer Y. Plants with double genomes might have had a better chance to survive the cretaceous–tertiary extinction event. Proc Natl Acad Sci. 2009;106:5737–42. 19325131 10.1073/pnas.0900906106 PMC2667025

[5] Chen R , Feng Z, Zhang X et al. A new way of rice breeding: Polyploid rice breeding. Plan Theory. 2021;10:422. 10.3390/plants10030422 PMC7996342 33668223

[6] Jansky SH , Spooner DM. The evolution of potato breeding. Plant Breed Rev. 2018;41:169–214.

[7] Jansky SH , Charkowski AO, Douches DS et al. Reinventing potato as a diploid inbred line-based crop. Crop Sci. 2016;56:1412–22.

[8] Parisod C , Holderegger R, Brochmann C. Evolutionary consequences of autopolyploidy. New Phytol. 2010;186:5–17. 20070540 10.1111/j.1469-8137.2009.03142.x

[9] Zhang H , Zhu B, Qi B et al. Evolution of the BBAA component of bread wheat during its history at the allohexaploid level. Plant Cell. 2014;26:2761–76. 24989045 10.1105/tpc.114.128439 PMC4145112

[10] Yang J , Liu D, Wang X et al. The genome sequence of allopolyploid *Brassica juncea* and analysis of differential homoeolog gene expression influencing selection. Nat Genet. 2016;48:1225–32. 27595476 10.1038/ng.3657

[11] Edger PP , Poorten TJ, VanBuren R et al. Origin and evolution of the octoploid strawberry genome. Nat Genet. 2019;51:541–7. 30804557 10.1038/s41588-019-0356-4 PMC6882729

[12] Chen ZJ , Sreedasyam A, Ando A et al. Genomic diversifications of five Gossypium allopolyploid species and their impact on cotton improvement. Nat Genet. 2020;52:525–33. 32313247 10.1038/s41588-020-0614-5 PMC7203012

[13] Li YP , Liu TJ, Luo HF et al. The transcriptional landscape of cultivated strawberry (*Fragaria× ananassa*) and its diploid ancestor (*Fragaria vesca*) during fruit development. J Integr Agric. 2021;20:1540–53.

[14] Auger DL , Gray AD, Ream TS et al. Nonadditive gene expression in diploid and triploid hybrids of maize. Genetics. 2005;169:389–97. 15489529 10.1534/genetics.104.032987 PMC1448873

[15] Song MJ , Potter BI, Doyle JJ et al. Gene balance predicts transcriptional responses immediately following ploidy change in Arabidopsis thaliana. Plant Cell. 2020;32:1434–48. 32184347 10.1105/tpc.19.00832 PMC7203931

[16] Chen P , Chen J, Sun M et al. Comparative transcriptome study of switchgrass (*Panicum virgatum* L.) homologous autopolyploid and its parental amphidiploid responding to consistent drought stress. Biotechnol Biofuels. 2020;13:170. 33072185 10.1186/s13068-020-01810-z PMC7559793

[17] Wang L , Cao S, Wang P et al. DNA hypomethylation in tetraploid rice potentiates stress-responsive gene expression for salt tolerance. Proc Natl Acad Sci. 2021;118:e2023981118. 33771925 10.1073/pnas.2023981118 PMC8020803

[18] Stupar RM , Bhaskar PB, Yandell BS et al. Phenotypic and transcriptomic changes associated with potato autopolyploidization. Genetics. 2007;176:2055–67. 17565939 10.1534/genetics.107.074286 PMC1950613

[19] Dai F , Wang Z, Luo G et al. Phenotypic and transcriptomic analyses of autotetraploid and diploid mulberry (*Morus alba* L.). Int J Mol Sci. 2015;16:22938–56. 26402678 10.3390/ijms160922938 PMC4613344

[20] Zhang X , Bernatavichute YV, Cokus S et al. Genome-wide analysis of mono-, di-and trimethylation of histone H3 lysine 4 in *Arabidopsis thaliana*. Genome Biol. 2009;10:R62–14. 19508735 10.1186/gb-2009-10-6-r62 PMC2718496

[21] Charron J-BF , He H, Elling AA et al. Dynamic landscapes of four histone modifications during deetiolation in Arabidopsis. Plant Cell. 2009;21:3732–48. 20008096 10.1105/tpc.109.066845 PMC2814509

[22] Zhang X , Clarenz O, Cokus S et al. Whole-genome analysis of histone H3 lysine 27 trimethylation in Arabidopsis. PLoS Biol. 2007;5:e129. 17439305 10.1371/journal.pbio.0050129 PMC1852588

[23] Sowokinos JR . Biochemical and molecular control of cold-induced sweetening in potatoes. Am J Potato Res. 2001;78:221–36.

[24] Bhaskar PB , Wu L, Busse JS et al. Suppression of the vacuolar invertase gene prevents cold-induced sweetening in potato. Plant Physiol. 2010;154:939–48. 20736383 10.1104/pp.110.162545 PMC2948980

[25] Bethke PC , Bussan AJ. Acrylamide in processed potato products. Am J Potato Res. 2013;90:403–24.

[26] Zhu X , Gong H, He Q et al. Silencing of vacuolar invertase and asparagine synthetase genes and its impact on acrylamide formation of fried potato products. Plant Biotechnol J. 2016;14:709–18. 26079224 10.1111/pbi.12421 PMC11388841

[27] Clasen BM , Stoddard TJ, Luo S et al. Improving cold storage and processing traits in potato through targeted gene knockout. Plant Biotechnol J. 2016;14:169–76. 25846201 10.1111/pbi.12370 PMC11389148

[28] Hameed A , Mehmood MA, Shahid M et al. Prospects for potato genome editing to engineer resistance against viruses and cold-induced sweetening. GM Crops Food. 2020;11:185–205. 31280681 10.1080/21645698.2019.1631115 PMC7518746

[29] Van Harsselaar JK , Lorenz J, Senning M et al. Genome-wide analysis of starch metabolism genes in potato (*Solanum tuberosum* L.). BMC Genomics. 2017;18:37. 10.1186/s12864-016-3381-z PMC5217216 28056783

[30] Liu J , Li J, Fu C. Comparative physiology and transcriptome analysis reveals the regulatory mechanism of genome duplication enhancing cold resistance in Fragaria nilgerrensis. Environ Exp Bot. 2021;188:104509.

[31] Hardigan MA , Crisovan E, Hamilton JP et al. Genome reduction uncovers a large dispensable genome and adaptive role for copy number variation in asexually propagated *Solanum tuberosum*. Plant Cell. 2016;28:388–405. 26772996 10.1105/tpc.15.00538 PMC4790865

[32] Potato Genome Sequencing Consortium . Genome sequence and analysis of the tuber crop potato. Nature. 2011;475:189–95. 21743474 10.1038/nature10158

[33] Karp A , Risiott R, Jones MG et al. Chromosome doubling in monohaploid and dihaploid potatoes by regeneration from cultured leaf explants. Plant Cell Tissue Organ Cult. 1984;3:363–73.

[34] de Alencar LD , Azevedo P, Latado RR. Mothers’ command: phenotypes changes resulting from reciprocal interploidy crosses. Euphytica. 2020;216:21.

[35] Miller M , Zhang C, Chen ZJ. Ploidy and hybridity effects on growth vigor and gene expression in *Arabidopsis thaliana* hybrids and their parents. *G3*. 2012;2:505–13. 10.1534/g3.112.002162 PMC3337479 22540042

[36] Saminathan T , Nimmakayala P, Manohar S et al. Differential gene expression and alternative splicing between diploid and tetraploid watermelon. J Exp Bot. 2015;66:1369–85. 25520388 10.1093/jxb/eru486 PMC4438448

[37] Hutten R , Schippers M, Hermsen J et al. Comparative performance of diploid and tetraploid progenies from 2x.2x crosses in potato. Euphytica. 1995;81:187–92.

[38] Maris B . Comparison of diploid and tetraploid potato families derived from *Solanum phureja* x dihaploid *S. tuberosum* hybrids and their vegetatively doubled counterparts. Euphytica. 1990;46:15–33.

[39] Ordoñez B . Assessment of ploidy by chloroplast count in stomatal guard cells. 2014. Lima (Peru). International Potato Center (CIP). 4 p.

[40] Braz GT , He L, Zhao H et al. Comparative oligo-FISH mapping: an efficient and powerful methodology to reveal karyotypic and chromosomal evolution. Genetics. 2018;208:513–23. 29242292 10.1534/genetics.117.300344 PMC5788518

[41] Hardigan MA , Laimbeer FPE, Newton L et al. Genome diversity of tuber-bearing solanum uncovers complex evolutionary history and targets of domestication in the cultivated potato. Proc Natl Acad Sci. 2017;114:E9999–10008. 29087343 10.1073/pnas.1714380114 PMC5699086

[42] Gong Z , Wu Y, Koblížková A et al. Repeatless and repeat-based centromeres in potato: implications for centromere evolution. Plant Cell. 2012;24:3559–74. 22968715 10.1105/tpc.112.100511 PMC3480287

[43] Gong L , Zhang H, Gan X et al. Transcriptome profiling of the potato (*Solanum tuberosum* L.) plant under drought stress and water-stimulus conditions. PLoS One. 2015;10:e0128041. 26010543 10.1371/journal.pone.0128041 PMC4444143

[44] Li Q , Qin Y, Hu X et al. Transcriptome analysis uncovers the gene expression profile of salt-stressed potato (*Solanum tuberosum* L.). Sci Rep. 2020;10:5411. 32214109 10.1038/s41598-020-62057-0 PMC7096413

[45] Hancock RD , Morris WL, Ducreux LJ et al. Physiological, biochemical and molecular responses of the potato (*Solanum tuberosum* L.) plant to moderately elevated temperature. Plant Cell Environ. 2014;37:439–50. 23889235 10.1111/pce.12168

[46] Mu H-Z , Liu Z-J, Lin L et al. Transcriptomic analysis of phenotypic changes in birch (*Betula platyphylla*) autotetraploids. Int J Mol Sci. 2012;13:13012–29. 23202935 10.3390/ijms131013012 PMC3497309

[47] Ruiz M , Oustric J, Santini J et al. Synthetic polyploidy in grafted crops. Front Plant Sci. 2020;11:1586. 10.3389/fpls.2020.540894 PMC7674608 33224156

[48] Liu Z , Jia Y, Ding Y et al. Plasma membrane CRPK1-mediated phosphorylation of 14-3-3 proteins induces their nuclear import to fine-tune CBF signaling during cold response. Mol Cell. 2017;66:117–128.e5. 28344081 10.1016/j.molcel.2017.02.016

[49] Kriegel A , Andrés Z, Medzihradszky A et al. Job sharing in the endomembrane system: vacuolar acidification requires the combined activity of V-ATPase and V-PPase. Plant Cell. 2015;27:3383–96. 26589552 10.1105/tpc.15.00733 PMC4707456

[50] Wang J , Lv J, Liu Z et al. Integration of transcriptomics and metabolomics for pepper (*Capsicum annuum* L.) in response to heat stress. Int J Mol Sci. 2019;20:5042. 31614571 10.3390/ijms20205042 PMC6829368

[51] Yang X , Zhu W, Zhang H et al. Heat shock factors in tomatoes: genome-wide identification, phylogenetic analysis and expression profiling under development and heat stress. PeerJ. 2016;4:e1961. 27190703 10.7717/peerj.1961 PMC4867723

[52] Trapero-Mozos A , Morris WL, Ducreux LJ et al. Engineering heat tolerance in potato by temperature-dependent expression of a specific allele of HEAT-SHOCK COGNATE 70. Plant Biotechnol J. 2018;16:197–207. 28509353 10.1111/pbi.12760 PMC5785350

[53] Bian XH , Li W, Niu CF et al. A class B heat shock factor selected for during soybean domestication contributes to salt tolerance by promoting flavonoid biosynthesis. New Phytol. 2020;225:268–83. 31400247 10.1111/nph.16104

[54] Stewart JJ , Demmig-Adams B, Cohu CM et al. Growth temperature impact on leaf form and function in *Arabidopsis thaliana* ecotypes from northern and southern Europe. Plant Cell Environ. 2016;39:1549–58. 26832121 10.1111/pce.12720

[55] Karimi R , Ershadi A, Rezaei Nejad A et al. Abscisic acid alleviates the deleterious effects of cold stress on ‘Sultana’grapevine (*Vitis vinifera* L.) plants by improving the anti-oxidant activity and photosynthetic capacity of leaves. J Hortic Sci Biotechnol. 2016;91:386–95.

[56] Oliveira G , Peñuelas J. Effects of winter cold stress on photosynthesis and photochemical efficiency of PSII of the Mediterranean Cistus albidus L. and Quercus ilex L. Plant Ecol. 2005;175:179–91.

[57] Basu P , Sharma A, Garg I et al. Tuber sink modifies photosynthetic response in potato under water stress. Environ Exp Bot. 1999;42:25–39.

[58] Zierer W , Rüscher D, Sonnewald U et al. Tuber and tuberous root development. Annu Rev Plant Biol. 2021;72:551–80. 33788583 10.1146/annurev-arplant-080720-084456

[59] Müller-Röber BT , Koßmann J, Hannah LC et al. One of two different ADP-glucose pyrophosphorylase genes from potato responds strongly to elevated levels of sucrose. Mol Gen Genet MGG. 1990;224:136–46. 1703626 10.1007/BF00259460

[60] Salanoubat M , Belliard G. The steady-state level of potato sucrose synthase mRN a is dependent on wounding, anaerobiosis and sucrose concentration. Gene. 1989;84:181–5. 2532612 10.1016/0378-1119(89)90153-4

[61] Liu X , Zhang C, Ou Y et al. Systematic analysis of potato acid invertase genes reveals that a cold-responsive member, StvacINV1, regulates cold-induced sweetening of tubers. Mol Gen Genomics. 2011;286:109–18. 10.1007/s00438-011-0632-1 21691778

[62] Zhang C , Yang Z, Tang D et al. Genome design of hybrid potato. Cell. 2021;184:3873–3883.e12. 34171306 10.1016/j.cell.2021.06.006

[63] Podwyszyńska M , Markiewicz M, Broniarek-Niemiec A et al. Apple autotetraploids with enhanced resistance to apple scab (*Venturia inaequalis*) due to genome duplication-phenotypic and genetic evaluation. Int J Mol Sci. 2021;22:527. 33430246 10.3390/ijms22020527 PMC7825683

[64] Zeng Z , Zhang T, Li G et al. Phenotypic and epigenetic changes occurred during the autopolyploidization of *Aegilops tauschii*. Cereal Res Commun. 2012;40:476–85.

[65] Xia J , Ma YJ, Wang Y et al. Deciphering transcriptome profiles of tetraploid *Artemisia annua* plants with high artemisinin content. Plant Physiol Biochem. 2018;130:112–26. 29982168 10.1016/j.plaphy.2018.06.018

[66] Yu Z , Haberer G, Matthes M et al. Impact of natural genetic variation on the transcriptome of autotetraploid Arabidopsis thaliana. Proc Natl Acad Sci. 2010;107:17809–14. 20876110 10.1073/pnas.1000852107 PMC2955106

[67] Onufriev AV , Schiessel H. The nucleosome: from structure to function through physics. Curr Opin Struct Biol. 2019;56:119–30. 30710748 10.1016/j.sbi.2018.11.003

[68] Allis CD , Jenuwein T. The molecular hallmarks of epigenetic control. Nat Rev Genet. 2016;17:487–500. 27346641 10.1038/nrg.2016.59

[69] Yan W , Chen D, Schumacher J et al. Dynamic control of enhancer activity drives stage-specific gene expression during flower morphogenesis. Nat Commun. 2019;10:1705. 30979870 10.1038/s41467-019-09513-2 PMC6461659

[70] Ricci WA , Lu Z, Ji L et al. Widespread long-range cis-regulatory elements in the maize genome. Nature Plants. 2019;5:1237–49. 31740773 10.1038/s41477-019-0547-0 PMC6904520

[71] Kato A , Lamb JC, Birchler JA. Chromosome painting using repetitive DNA sequences as probes for somatic chromosome identification in maize. Proc Natl Acad Sci. 2004;101:13554–9. 15342909 10.1073/pnas.0403659101 PMC518793

[72] Han Y , Zhang T, Thammapichai P et al. Chromosome-specific painting in Cucumis species using bulked oligonucleotides. Genetics. 2015;200:771–9. 25971668 10.1534/genetics.115.177642 PMC4512542

[73] He L , Zhao H, He J et al. Extraordinarily conserved chromosomal synteny of citrus species revealed by chromosome-specific painting. Plant J. 2020;103:2225–35. 32578280 10.1111/tpj.14894

[74] Ye J , Shakya R, Shrestha P et al. Tuber-specific silencing of the acid invertase gene substantially lowers the acrylamide-forming potential of potato. J Agric Food Chem. 2010;58:12162–7. 21049996 10.1021/jf1032262

[75] Zeng Z , Zhang W, Marand AP et al. Cold stress induces enhanced chromatin accessibility and bivalent histone modifications H3K4me3 and H3K27me3 of active genes in potato. Genome Biol. 2019;20:123. 31208436 10.1186/s13059-019-1731-2 PMC6580510

[76] Chen S , Zhou Y, Chen Y et al. Fastp: an ultra-fast all-in-one FASTQ preprocessor. Bioinformatics. 2018;34:i884–90. 30423086 10.1093/bioinformatics/bty560 PMC6129281

[77] Kim D , Paggi JM, Park C et al. Graph-based genome alignment and genotyping with HISAT2 and HISAT-genotype. Nat Biotechnol. 2019;37:907–15. 31375807 10.1038/s41587-019-0201-4 PMC7605509

[78] Pertea M , Pertea GM, Antonescu CM et al. StringTie enables improved reconstruction of a transcriptome from RNA-seq reads. Nat Biotechnol. 2015;33:290–5. 25690850 10.1038/nbt.3122 PMC4643835

[79] Love MI , Huber W, Anders S. Moderated estimation of fold change and dispersion for RNA-seq data with DESeq2. Genome Biol. 2014;15:550. 25516281 10.1186/s13059-014-0550-8 PMC4302049

[80] Trapnell C , Williams BA, Pertea G et al. Transcript assembly and quantification by RNA-Seq reveals unannotated transcripts and isoform switching during cell differentiation. Nat Biotechnol. 2010;28:511–5. 20436464 10.1038/nbt.1621 PMC3146043

[81] Li H , Durbin R. Fast and accurate short read alignment with burrows–wheeler transform. Bioinformatics. 2009;25:1754–60. 19451168 10.1093/bioinformatics/btp324 PMC2705234

[82] Zhang Y , Liu T, Meyer CA et al. Model-based analysis of ChIP-Seq (MACS). Genome Biol. 2008;9:R137. 18798982 10.1186/gb-2008-9-9-r137 PMC2592715

[83] Tian T , Liu Y, Yan H et al. agriGO v2.0: a GO analysis toolkit for the agricultural community, 2017 update. Nucleic Acids Res. 2017;45:W122–9. 28472432 10.1093/nar/gkx382 PMC5793732

[84] Li H . A statistical framework for SNP calling, mutation discovery, association mapping and population genetical parameter estimation from sequencing data. Bioinformatics. 2011;27:2987–93. 21903627 10.1093/bioinformatics/btr509 PMC3198575

[85] Rausch T , Zichner T, Schlattl A et al. DELLY: structural variant discovery by integrated paired-end and split-read analysis. Bioinformatics. 2012;28:i333–9. 22962449 10.1093/bioinformatics/bts378 PMC3436805

