# Supplementary material for: Development of homozygous tetraploid potato and whole genome doubling-induced the enrichment of H3K27ac and potentially enhanced resistance to cold-induced sweetening in tubers
Source: Hortic Res. 2023 Feb 8;10(3):uhad017. doi: 10.1093/hr/uhad017 (PMC10031744; doi:10.1093/hr/uhad017)
Supplement: Web_Material_uhad017 [file web_material_uhad017.zip › STable.docx]

| **Table S1 Statistics of phenotypes for the diploid and tetraploid potato lines** | | | |  |
| --- | --- | --- | --- | --- |
| **Phenotype** | **DM2X-new** | **DM4X-13** | **DM4X-17** |  |
| Plant architecture | dispersal | upright | upright |  |
| Plant height (cm) | 32±3.65 | 35.83±3.34 | 40.83±3.58** |  |
| Branch number | 32.83±2.34 | 12±1.29*** | 11.33±1.70*** |  |
| Stem diameter (mm) | 4.67±0.47 | 6.83±0.69*** | 7.5±0.5*** |  |
| Leaf area | 2.54±1.53 | 3.45±2.21 | 4.32±2.47*** |  |
| Corolla color | white | white | white |  |
| Tuber yield (g) | 48.37±10.91 | 13.61±7.38*** | 21.72±9.07** |  |
| Tuber number | 34±10.07 | 8.17±3.34*** | 6.83±2.34*** |  |
| Average tuber weight (g) | 1.48±0.42 | 1.72±0.74 | 3.35±1.21** |  |
| Tuber length (cm) | 2.92±1.35 | 3.17±1.63 | 4.04±1.86*** |  |
| Tuber width (cm) | 0.87±0.27 | 0.92±0.25 | 1.07±0.29*** |  |
| Note: Each value includes mean ± standard deviation of 6 biological replicates. Differences in means between the tetraploid lines and the diploid line are indicated as significance at *p*<0.01 (**), or *p*<0.001 (***) level. | | | |  |
|  |  |  |  |  |
|  |  |  |  |  |

| **Table S2 Genome re-sequencing of DM2X-new, DM4X-13 and DM4X-17.** | | | | | |
| --- | --- | --- | --- | --- | --- |
| **Plant name** | **Raw PE read #** | **Read # after QC** | **Mapped read #** | **Mapped read %** | **Genome coverage (X)** |
| DM2X-new | 68,187,098 | 67,729,992 | 60,155,064 | 88.82% | 20.4X |
| DM4X-13 | 101,843,145 | 101,054,401 | 91,097,037 | 90.15% | 30.9X |
| DM4X-17 | 106,631,881 | 105,816,560 | 95,215,242 | 89.98% | 32.3X |

| **Table S3. RNA-seq data generated from RT and cold tubers of DM2X-new, DM4X-13 and DM4X-17.** | | | | |
| --- | --- | --- | --- | --- |
| **Plant name** | **Treatment** | **Biological replicate** | **Mapped PE reads #** | **Uniquely mapped PE reads #** |
| DM2X-new | RT for 14 days | rep1 | 32,652,531 | 24,582,448 |
| DM2X-new | RT for 14 days | rep2 | 26,172,657 | 29,566,461 |
| DM2X-new | 4℃ for 14 days | rep1 | 14,898,035 | 18,014,246 |
| DM2X-new | 4℃ for 14 days | rep2 | 19,994,318 | 14,025,849 |
| DM4X-13 | RT for 14 days | rep1 | 15,384,586 | 14,047,943 |
| DM4X-13 | RT for 14 days | rep2 | 16,198,464 | 15,181,007 |
| DM4X-13 | 4℃ for 14 days | rep1 | 16,054,772 | 14,622,882 |
| DM4X-13 | 4℃ for 14 days | rep2 | 23,059,575 | 18,564,489 |
| DM4X-17 | RT for 14 days | rep1 | 17,558,517 | 16,098,698 |
| DM4X-17 | RT for 14 days | rep2 | 22,814,768 | 21,238,986 |
| DM4X-17 | 4℃ for 14 days | rep1 | 26,080,363 | 24,251,010 |
| DM4X-17 | 4℃ for 14 days | rep2 | 31,426,732 | 28,968,832 |

| **Table S4. ChIP-seq data generated from RT and cold tubers of DM2X-new, DM4X-13 and DM4X-17.** | | | | | |
| --- | --- | --- | --- | --- | --- |
| **Plant name** | **Treatment** | **Antibody** | **Mapped PE reads #** | **Uniquely mapped PE reads #** |  |
| DM2X-new | RT for 14 days | H3K4me3 | 15,945,456 | 11,039,983 |  |
| DM2X-new | RT for 14 days | H3K27me3 | 40,077,520 | 25,685,113 |  |
| DM2X-new | RT for 14 days | H3K27ac | 39,819,087 | 28,656,285 |  |
| DM2X-new | RT for 14 days | IgG | 19,669,769 | 10,918,566 |  |
| DM2X-new | 4℃ for 14 days | H3K4me3 | 43,189,684 | 31,949,940 |  |
| DM2X-new | 4℃ for 14 days | H3K27me3 | 36,750,539 | 25,382,068 |  |
| DM2X-new | 4℃ for 14 days | H3K27ac | 39,187,739 | 21,191,435 |  |
| DM2X-new | 4℃ for 14 days | IgG | 37,126,442 | 24,635,390 |  |
| DM4X-13 | RT for 14 days | H3K4me3 | 30,921,302 | 20,748,230 |  |
| DM4X-13 | RT for 14 days | H3K27me3 | 36,501,507 | 23,261,155 |  |
| DM4X-13 | RT for 14 days | H3K27ac | 41,507,820 | 26,076,855 |  |
| DM4X-13 | RT for 14 days | IgG | 20,814,301 | 9,110,469 |  |
| DM4X-13 | 4℃ for 14 days | H3K4me3 | 33,842,394 | 25,340,709 |  |
| DM4X-13 | 4℃ for 14 days | H3K27me3 | 41,361,451 | 27,575,159 |  |
| DM4X-13 | 4℃ for 14 days | H3K27ac | 37,091,299 | 27,215,668 |  |
| DM4X-13 | 4℃ for 14 days | IgG | 26,692,195 | 18,081,864 |  |
| DM4X-17 | RT for 14 days | H3K4me3 | 29,194,133 | 19,412,758 |  |
| DM4X-17 | RT for 14 days | H3K27me3 | 38,460,503 | 25,890,684 |  |
| DM4X-17 | RT for 14 days | H3K27ac | 33,657,475 | 24,357,012 |  |
| DM4X-17 | RT for 14 days | IgG | 20,111,641 | 5,631,825 |  |
| DM4X-17 | 4℃ for 14 days | H3K4me3 | 43,650,833 | 32,975,938 |  |
| DM4X-17 | 4℃ for 14 days | H3K27me3 | 33,940,649 | 22,782,105 |  |
| DM4X-17 | 4℃ for 14 days | H3K27ac | 35,243,398 | 23,336,537 |  |
| DM4X-17 | 4℃ for 14 days | IgG | 30,443,703 | 20,053,438 |  |

**Table S5. The expression of CIS-related genes in RT and cold tubers of DM2X-new, DM4X-13 and DM4X-17.**

| **Gene ID** | **Gene name** | **DM2X-new (TPM)** | **Cold DM2X-new (TPM)** | **log2FC(cold/RT DM2X-new)** | **DM4X-13 (TPM)** | **Cold DM4X-13 (TPM)** | **log2FC(cold/RT DM4X-13)** | **DM4X-17 (TPM)** | **Cold DM4X-17 (TPM)** | **log2FC(cold/RT DM4X-17)** |
| --- | --- | --- | --- | --- | --- | --- | --- | --- | --- | --- |
| PGSC0003DMG400001912 | *PGM* | 30.5955 | 29.4616 | -0.054483694 | 27.9226 | 31.258 | 0.162792189 | 24.4816 | 29.1293 | 0.250773184 |
| PGSC0003DMG400031084 | *AGPS1.1* | 736.664 | 167.116 | -2.140156875 | 651.627 | 129.535 | -2.33070442 | 575.194 | 690.646 | 0.2638978 |
| PGSC0003DMG400000735 | *AGPL3* | 680.379 | 112.669 | -2.594247989 | 537.317 | 60.7894 | -3.143881799 | 571.221 | 641.637 | 0.167708319 |
| PGSC0003DMG400015952 | *AGPL2* | 24.143 | 42.873 | 0.828464416 | 24.5579 | 43.6549 | 0.829956399 | 28.5953 | 40.3598 | 0.497140984 |
| PGSC0003DMG400012111 | *Gbss1* | 1003.44 | 275.571 | -1.864458377 | 938.923 | 146.654 | -2.678590428 | 923.725 | 972.784 | 0.074656086 |
| PGSC0003DMG400001328 | *Gbss2* | 48.262 | 18.387 | -1.392201592 | 44.3963 | 14.2316 | -1.64134158 | 39.4831 | 36.374 | -0.118327681 |
| PGSC0003DMG402018552 | *SS1* | 6.36307 | 9.08719 | 0.514111251 | 6.95542 | 6.95615 | 0.000151409 | 7.39607 | 13.7349 | 0.893015622 |
| PGSC0003DMG400001328 | *SS2* | 48.262 | 18.387 | -1.392201592 | 44.3963 | 14.2316 | -1.64134158 | 39.4831 | 36.374 | -0.118327681 |
| PGSC0003DMG400008322 | *SS4* | 2.99998 | 9.19244 | 1.615494972 | 2.2936 | 7.9497 | 1.793286608 | 2.35874 | 5.36292 | 1.185002331 |
| PGSC0003DMG400016481 | *SS3* | 66.6966 | 45.4142 | -0.554469752 | 53.2663 | 40.1057 | -0.409415778 | 52.2998 | 120.808 | 1.20783866 |
| PGSC0003DMG400030619 | *SS5* | 23.738 | 5.31246 | -2.15974641 | 26.2545 | 3.46622 | -2.921129591 | 18.0683 | 13.2009 | -0.452824481 |
| PGSC0003DMG401013540 | *SS6* | 24.607 | 17.0789 | -0.526853722 | 24.5176 | 11.7427 | -1.062053597 | 23.2228 | 16.3493 | -0.506313062 |
| PGSC0003DMG402007274 | *DBE6* | 223.57 | 70.325 | -1.668617059 | 230.703 | 58.3288 | -1.983756466 | 212.193 | 76.5054 | -1.471743578 |
| PGSC0003DMG400020699 | *DBE7* | 167.81 | 28.1352 | -2.576380566 | 137.475 | 16.7366 | -3.038090904 | 124.353 | 56.6222 | -1.135001603 |
| PGSC0003DMG400000954 | *DBE9* | 53.4601 | 24.4602 | -1.128026336 | 51.1817 | 18.9697 | -1.431931204 | 37.2452 | 23.8944 | -0.640381968 |
| PGSC0003DMG400009981 | *SBE* | 547.386 | 460.138 | -0.250491932 | 559.551 | 342.579 | -0.707832914 | 509.199 | 1141.16 | 1.164199592 |
| PGSC0003DMG400002479 | *PHO1a.1* | 8423.71 | 522.688 | -4.010433827 | 5189.79 | 371.385 | -3.80468871 | 6778.35 | 2349 | -1.528887419 |
| PGSC0003DMG400003495 | *PHO1a.2* | 1670.49 | 144.301 | -3.533118144 | 1177.41 | 104.871 | -3.488929097 | 1006.74 | 377.089 | -1.41671417 |
| PGSC0003DMG400007782 | *PHO1a.3* | 2484.13 | 163.781 | -3.922900768 | 1880.37 | 94.8681 | -4.308949705 | 1661.93 | 497.315 | -1.740627766 |
| PGSC0003DMG400033858 | *PHO1a.4* | 526.633 | 49.4262 | -3.413450029 | 363.036 | 30.5795 | -3.569475896 | 318.775 | 138.575 | -1.201871482 |
| PGSC0003DMG400015246 | *SEX4* | 224.708 | 301.587 | 0.424522754 | 243.617 | 142.782 | -0.770800695 | 185.232 | 283.515 | 0.614091711 |
| PGSC0003DMG400027327 | *SEX4-like* | 521.186 | 518.11 | -0.008539902 | 407.425 | 868.448 | 1.091904959 | 358.117 | 624.424 | 0.802094983 |
| PGSC0003DMG400030092 | *LSF1* | 48.9052 | 58.9225 | 0.268830771 | 55.4923 | 47.7981 | -0.215334328 | 51.7906 | 41.4386 | -0.32171501 |
| PGSC0003DMG400029073 | *LSF2* | 63.7725 | 39.4597 | -0.692554451 | 58.4751 | 22.5095 | -1.377288413 | 53.7355 | 24.0075 | -1.162390332 |
| PGSC0003DMG400007974 | *AMY 1.1* | 0.820653 | 2.83824 | 1.790152353 | 0.678242 | 4.38923 | 2.69409584 | 0.454086 | 1.55221 | 1.773286292 |
| PGSC0003DMG400009891 | *AMY 2* | 90.7392 | 28.325 | -1.679649984 | 79.4469 | 28.6435 | -1.471783138 | 72.9982 | 61.873 | -0.238550903 |
| PGSC0003DMG401017626 | *AMY 3* | 13.0585 | 29.6088 | 1.181036834 | 17.7069 | 27.9468 | 0.658371442 | 17.7234 | 18.7725 | 0.082965399 |
| PGSC0003DMG400016589 | *DPE1* | 145.74 | 76.438 | -0.93103496 | 171.536 | 55.9195 | -1.617088018 | 124.752 | 127.057 | 0.026412916 |
| PGSC0003DMG400001549 | *BAM 1* | 36.4663 | 191.384 | 2.391834492 | 41.8932 | 233.194 | 2.476742676 | 49.7937 | 107.917 | 1.115887022 |
| PGSC0003DMG400001855 | *BAM 3.1* | 1.68649 | 63.1609 | 5.22693606 | 1.75095 | 114.843 | 6.035381226 | 3.29541 | 32.3604 | 3.295699566 |
| PGSC0003DMG400010664 | *BAM 9* | 306.299 | 789.15 | 1.365358894 | 393.518 | 725.664 | 0.882872072 | 429.81 | 440.715 | 0.036146951 |
| PGSC0003DMG400012129 | *BAM 4* | 29.8094 | 18.4379 | -0.693092989 | 34.6475 | 18.7281 | -0.887546715 | 28.825 | 17.6503 | -0.707627903 |
| PGSC0003DMG400013856 | *Vinv* | 15.3901 | 265.986 | 4.111275801 | 25.9632 | 178.824 | 2.78400026 | 14.3224 | 72.2606 | 2.334935969 |
| PGSC0003DMG400013547 | *Susy1* | 1.66675 | 0.334433 | -2.31724861 | 2.44626 | 0 | -inf | 1.86313 | 0.793525 | -1.231380761 |
| PGSC0003DMG400013546 | *Susy2* | 105.222 | 192.982 | 0.875029912 | 131.029 | 259.768 | 0.987337569 | 116.927 | 125.281 | 0.099559528 |
| PGSC0003DMG400006672 | *Susy3* | 50.6512 | 102.52 | 1.01723703 | 91.0323 | 67.7462 | -0.426238506 | 79.8505 | 91.528 | 0.196911715 |
| PGSC0003DMG400002895 | *Susy4* | 1366.19 | 345.931 | -1.981601928 | 1329.28 | 201.011 | -2.725298669 | 1482.47 | 3472.35 | 1.227909463 |
| PGSC0003DMG400031046 | *Susy6* | 1.46704 | 11.1697 | 2.928610325 | 0.785358 | 3.01019 | 1.9384322 | 3.69053 | 12.7083 | 1.78387113 |
| PGSC0003DMG400016730 | *Susy7* | 1.04793 | 10.5447 | 3.330903795 | 0.707315 | 5.00561 | 2.823121129 | 2.70743 | 13.671 | 2.336122834 |
| PGSC0003DMG401031123 | *UGPase1* | 18.3094 | 28.3028 | 0.628360271 | 19.237 | 31.9955 | 0.733985183 | 11.993 | 22.0568 | 0.879030912 |
| PGSC0003DMG401013333 | *UGPase2* | 1514.5 | 952.614 | -0.668877922 | 1504.24 | 686.844 | -1.130980398 | 1243.29 | 971.871 | -0.35532611 |
| PGSC0003DMG400001912 | *PGM1* | 30.5955 | 29.4616 | -0.054483694 | 27.9226 | 31.258 | 0.162792189 | 24.4816 | 29.1293 | 0.250773184 |
| PGSC0003DMG400005269 | *GPT2.1* | 545.342 | 12.6633 | -5.428435951 | 447.547 | 3.5433 | -6.980801768 | 480.416 | 438.34 | -0.132233864 |

| **Table S6. Primers used for qRT-PCR.** | | | |
| --- | --- | --- | --- |
| **Gene name** | **Gene ID** | **Forward** | **Reverse** |
| *GPT2.1* | PGSC0003DMG400005269 | GATTCCCTTTGCTATTGCTGTG | GCCACCACCCACCATATAAA |
| *VINV* | PGSC0003DMG400013856 | GAGTACTGTGGGGATGGATTG | TCAACTGGCCACTGAAGTAGAT |
| *SuSy4* | PGSC0003DMG400002895 | CTCATGAGCTTTTGGCTGAG | CTCAAACGAATAGCAAGTGCA |
| *EF1α* | PGSC0003DMG400023270 | ATTGGAAACGGATATGCTCCA | TCCTTACCTGAACGCCTGTCA |
