# Supplementary figures and images for: Development of homozygous tetraploid potato and whole genome doubling-induced the enrichment of H3K27ac and potentially enhanced resistance to cold-induced sweetening in tubers
Source: Hortic Res. 2023 Feb 8;10(3):uhad017. doi: 10.1093/hr/uhad017 (PMC10031744; doi:10.1093/hr/uhad017)

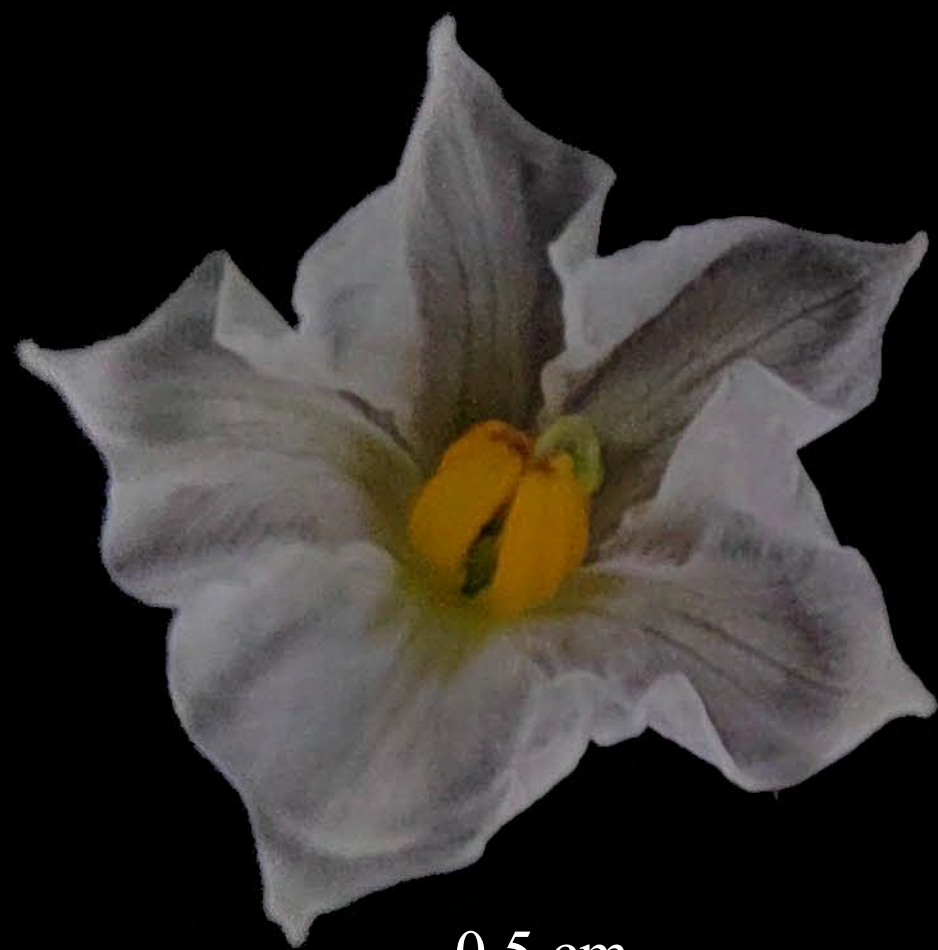

0.5 cm

DM2X-new

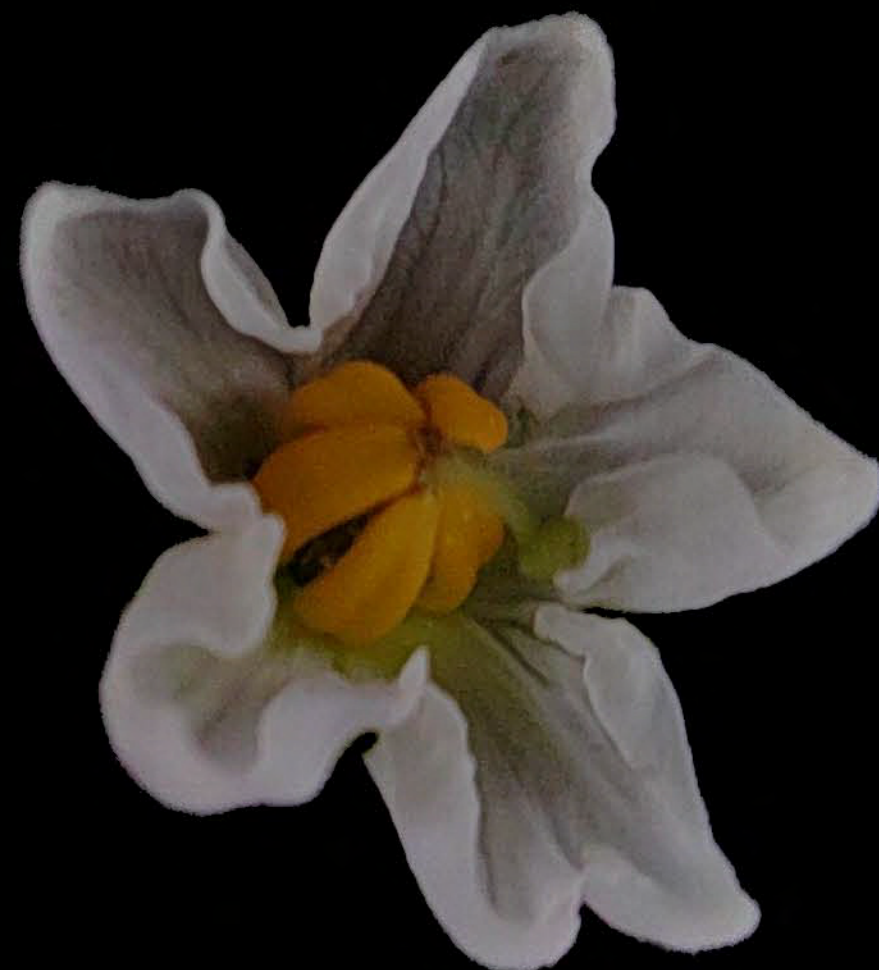

0.5 cm

DM4X-13

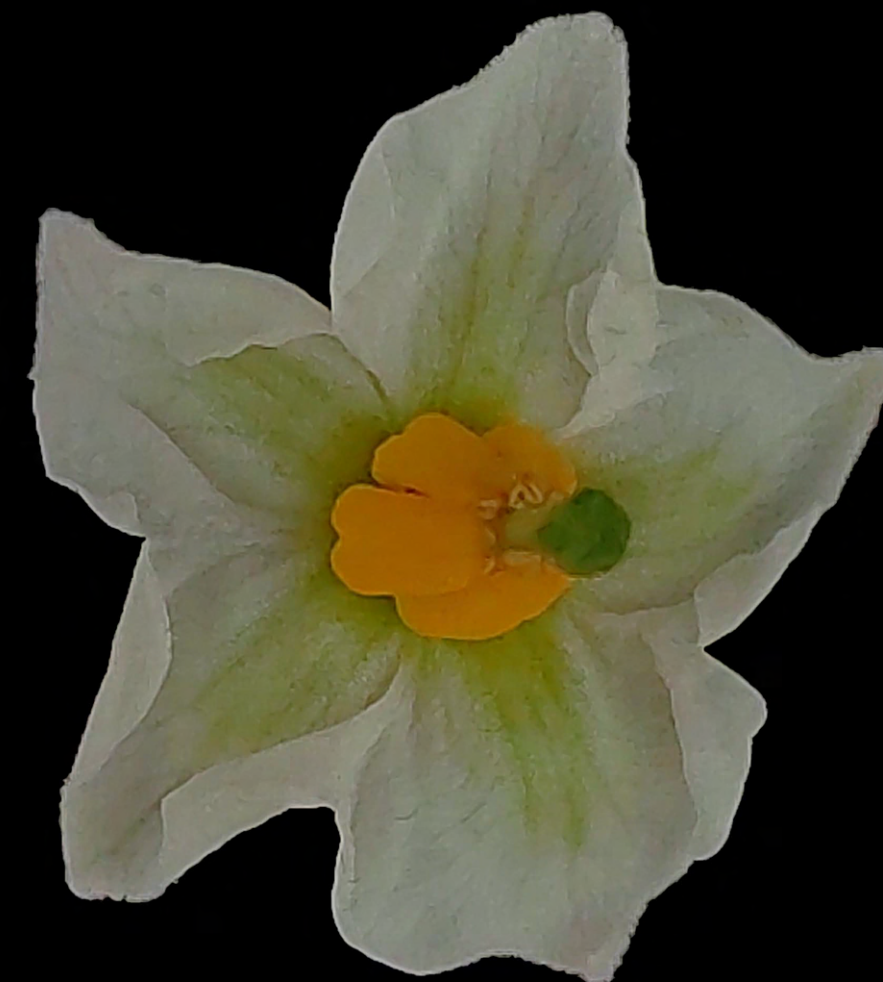

0.5 cm

DM4X-17

**Figure S1**

Supplement: Web_Material_uhad017 [file web_material_uhad017.zip › Figure S1.pdf]

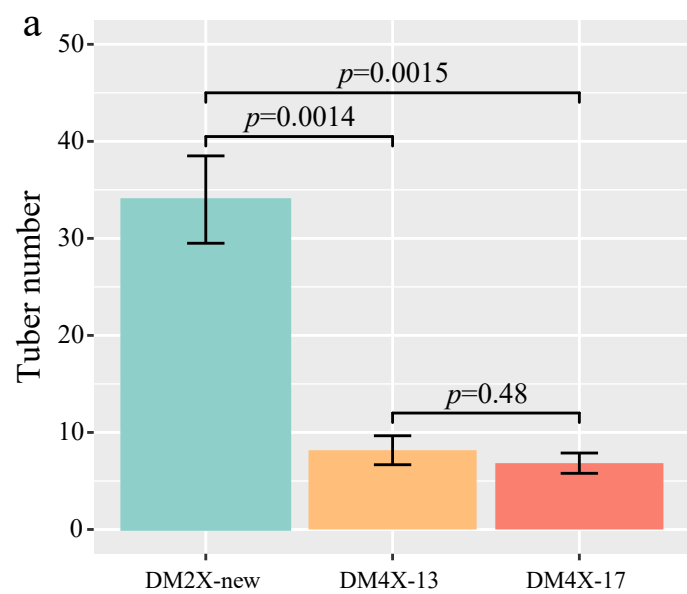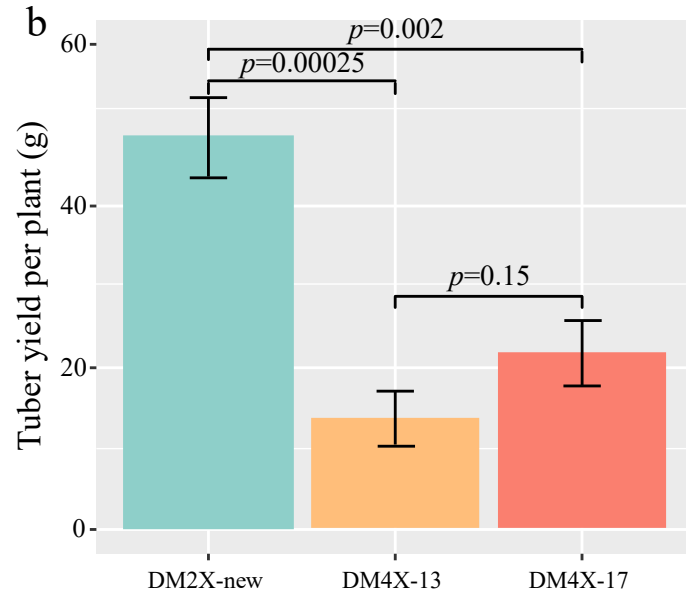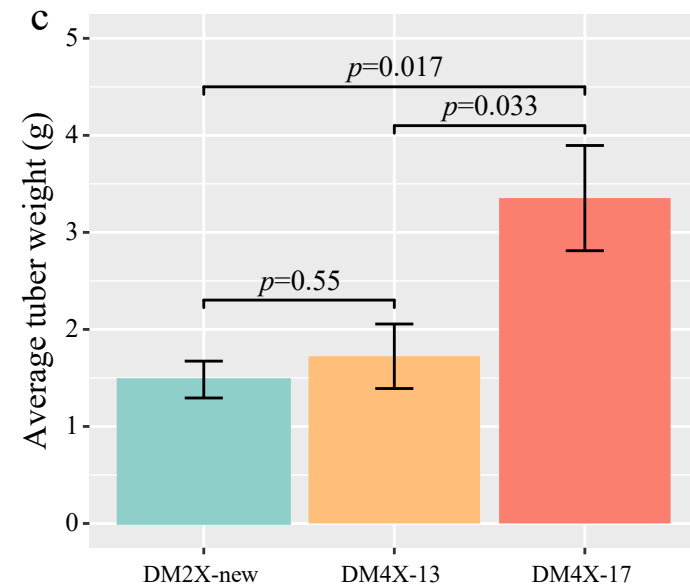

**Figure S2**

Supplement: Web_Material_uhad017 [file web_material_uhad017.zip › Figure S2.pdf]

a

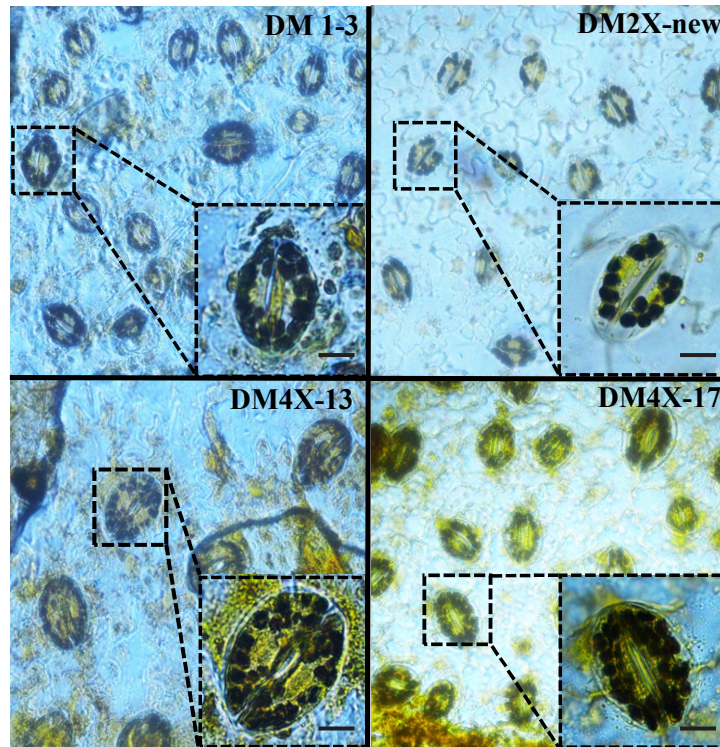

b

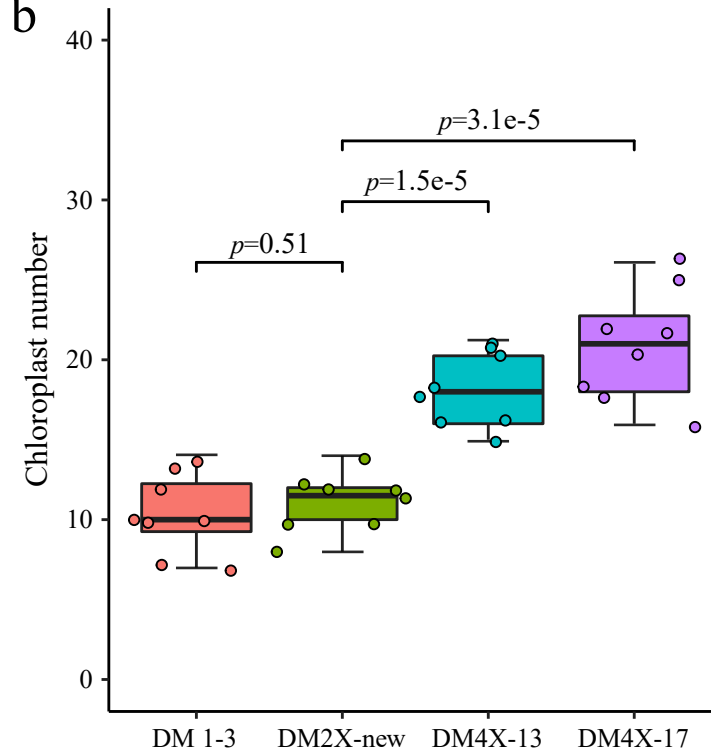

Figure S3

Supplement: Web_Material_uhad017 [file web_material_uhad017.zip › Figure S3.pdf]

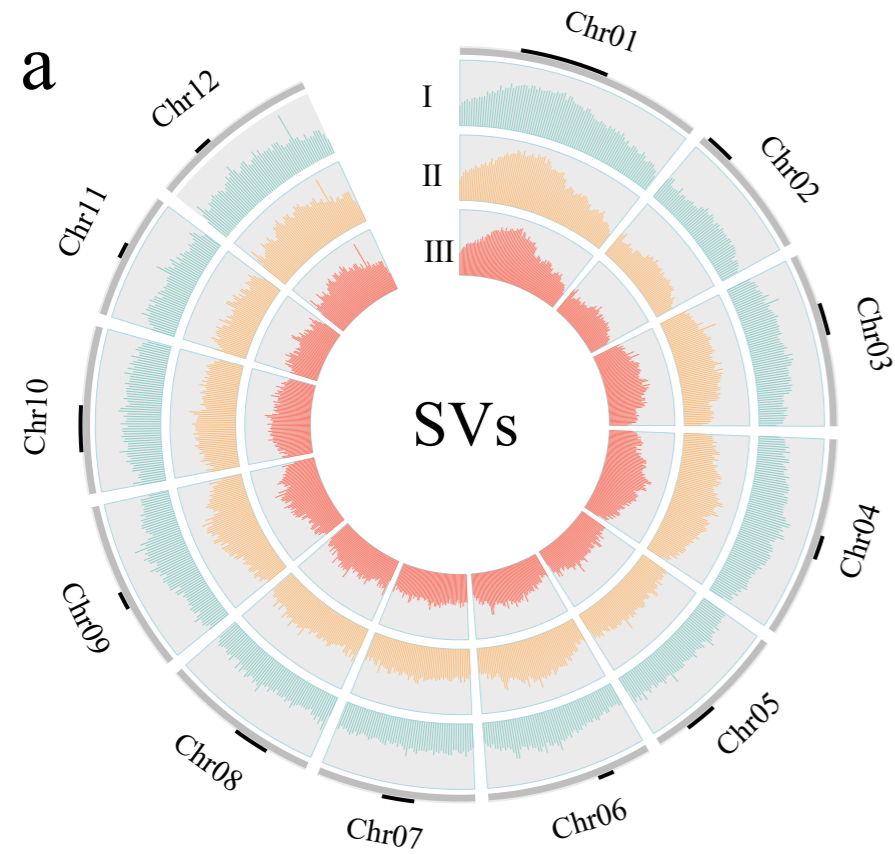

|             |        |
|-------------|--------|
| I DM2X-new  | 11,217 |
| II DM4X-13  | 14,729 |
| III DM4X-17 | 15,836 |

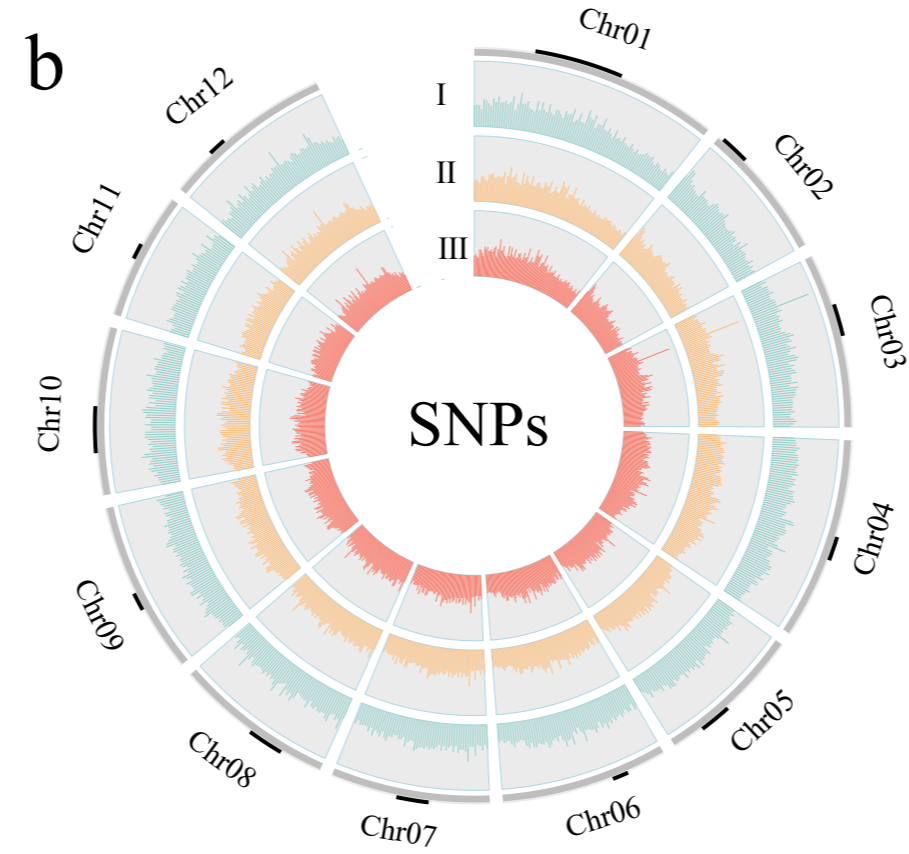

|             |         |
|-------------|---------|
| I DM2X-new  | 195,121 |
| II DM4X-13  | 320,912 |
| III DM4X-17 | 333,038 |

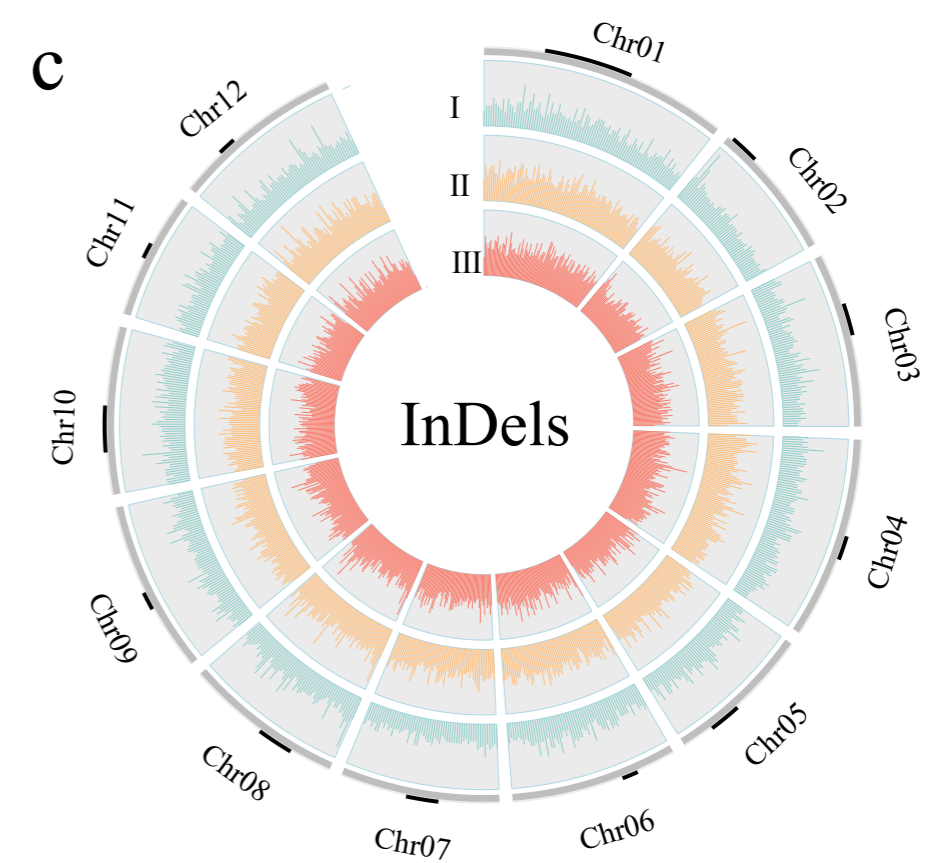

|             |        |
|-------------|--------|
| I DM2X-new  | 2,128  |
| II DM4X-13  | 10,521 |
| III DM4X-17 | 11,513 |

**Figure S4**

Supplement: Web_Material_uhad017 [file web_material_uhad017.zip › Figure S4.pdf]

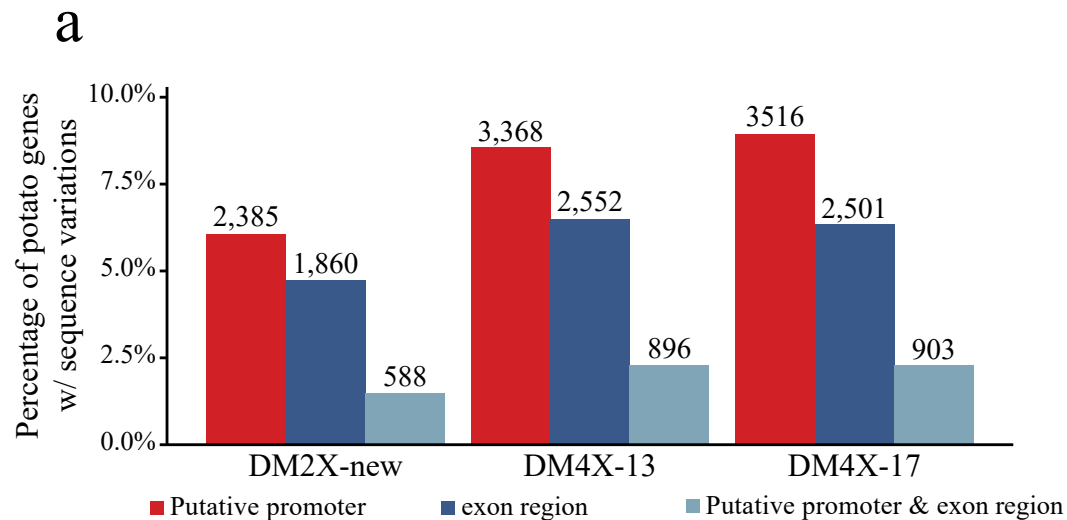

**b** Genes w/ sequence variations

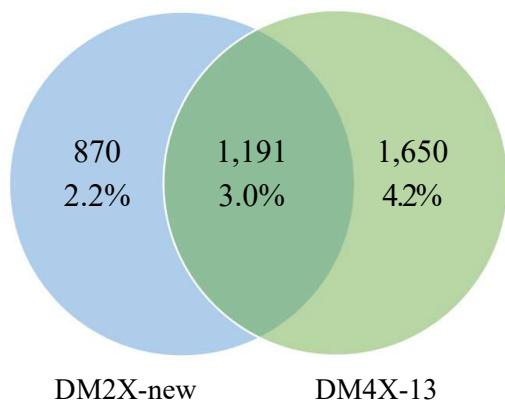

**c** Genes w/ sequence variations

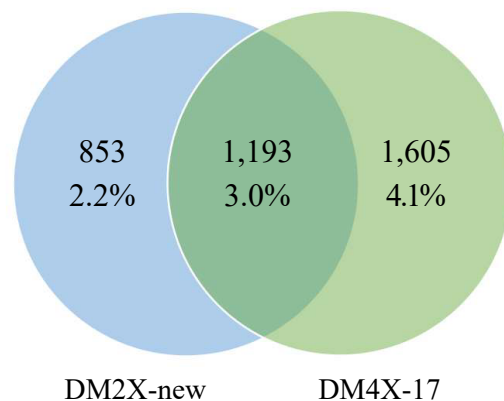

**Figure S5**

Supplement: Web_Material_uhad017 [file web_material_uhad017.zip › Figure S5.pdf]

a

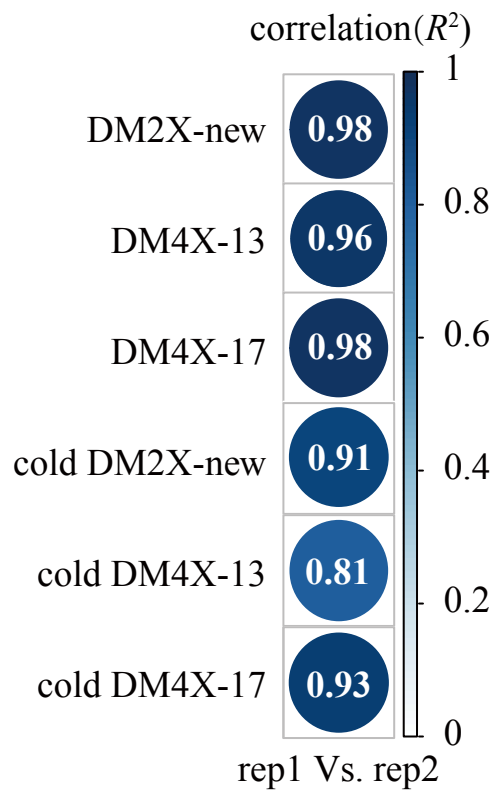

b

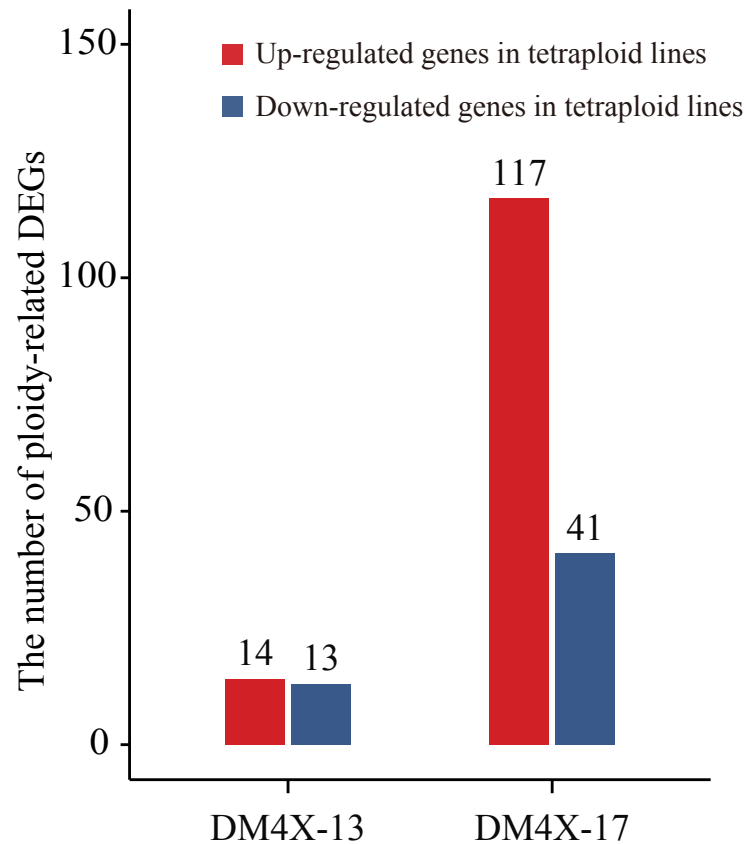

Figure S6

Supplement: Web_Material_uhad017 [file web_material_uhad017.zip › Figure S6.pdf]

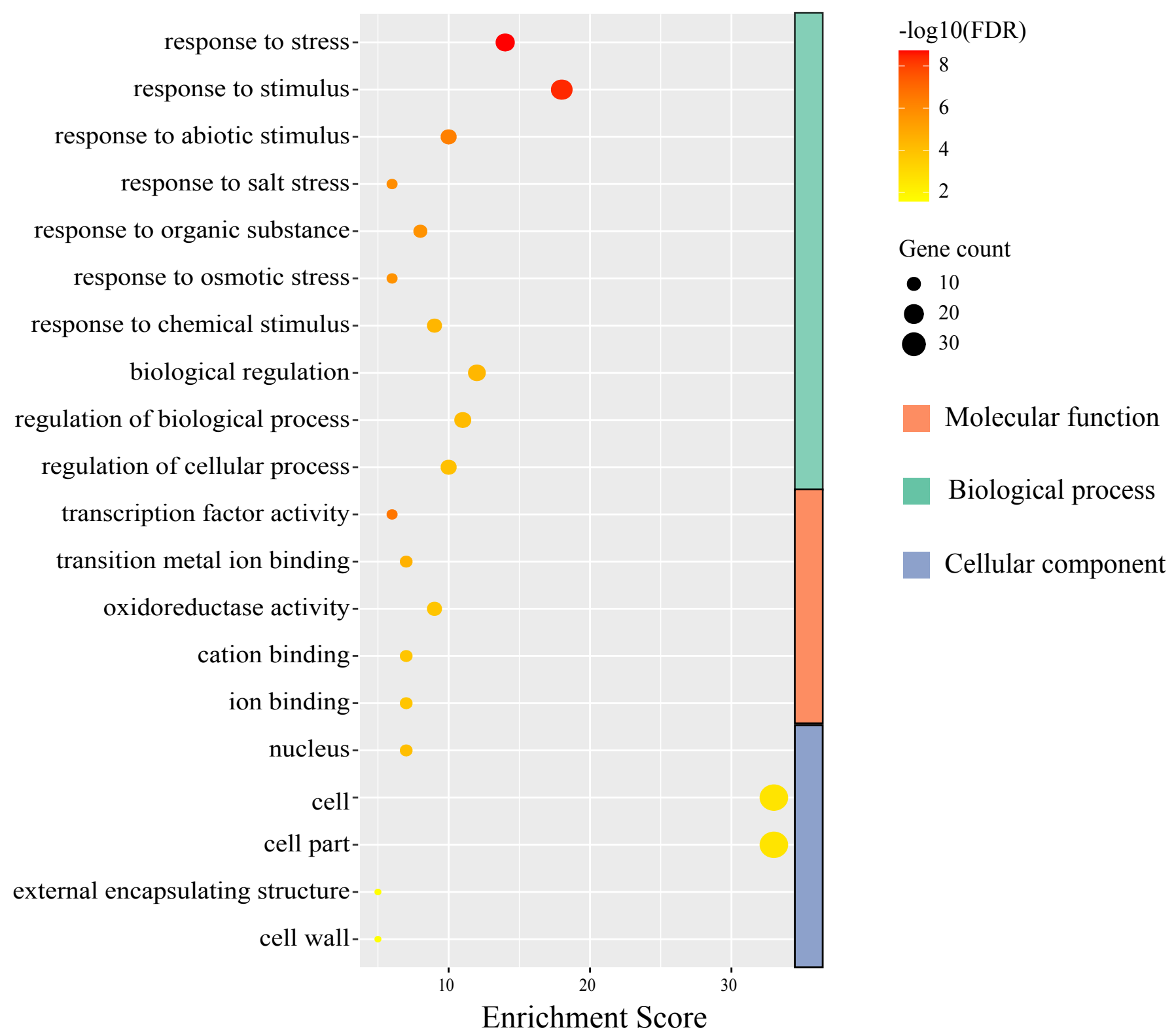

**Figure S7**

Supplement: Web_Material_uhad017 [file web_material_uhad017.zip › Figure S7.pdf]

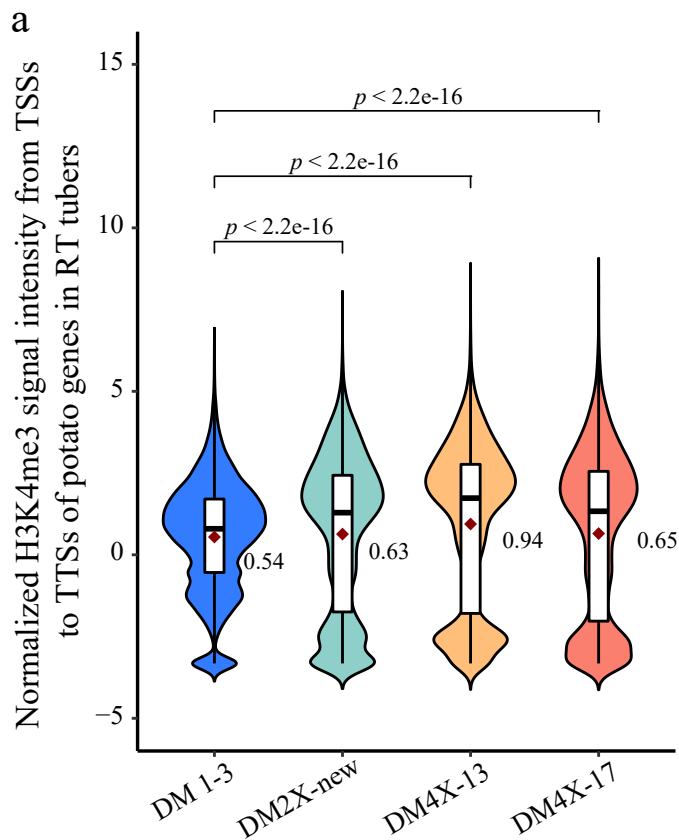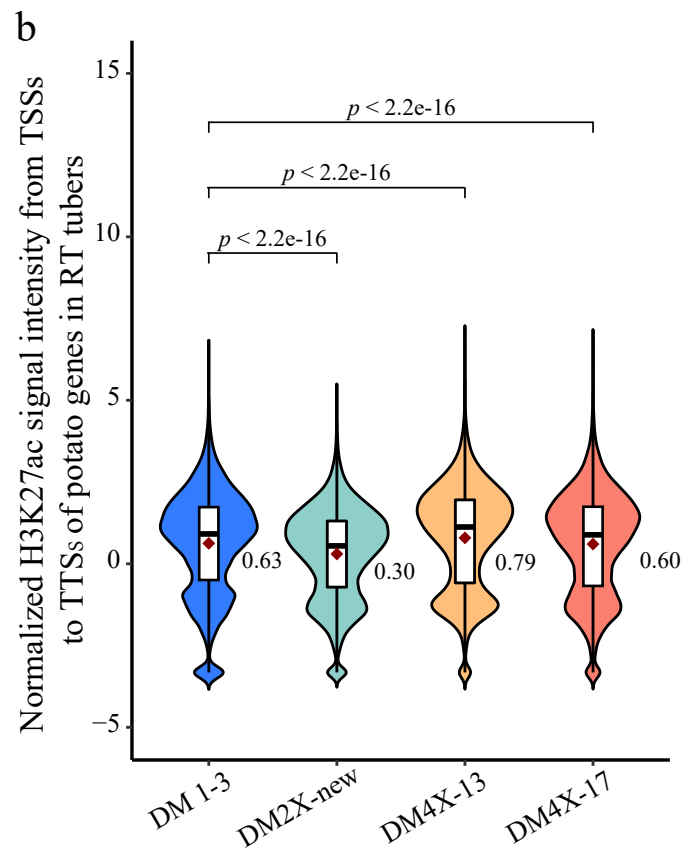

**Figure S8**

Supplement: Web_Material_uhad017 [file web_material_uhad017.zip › Figure S8.pdf]

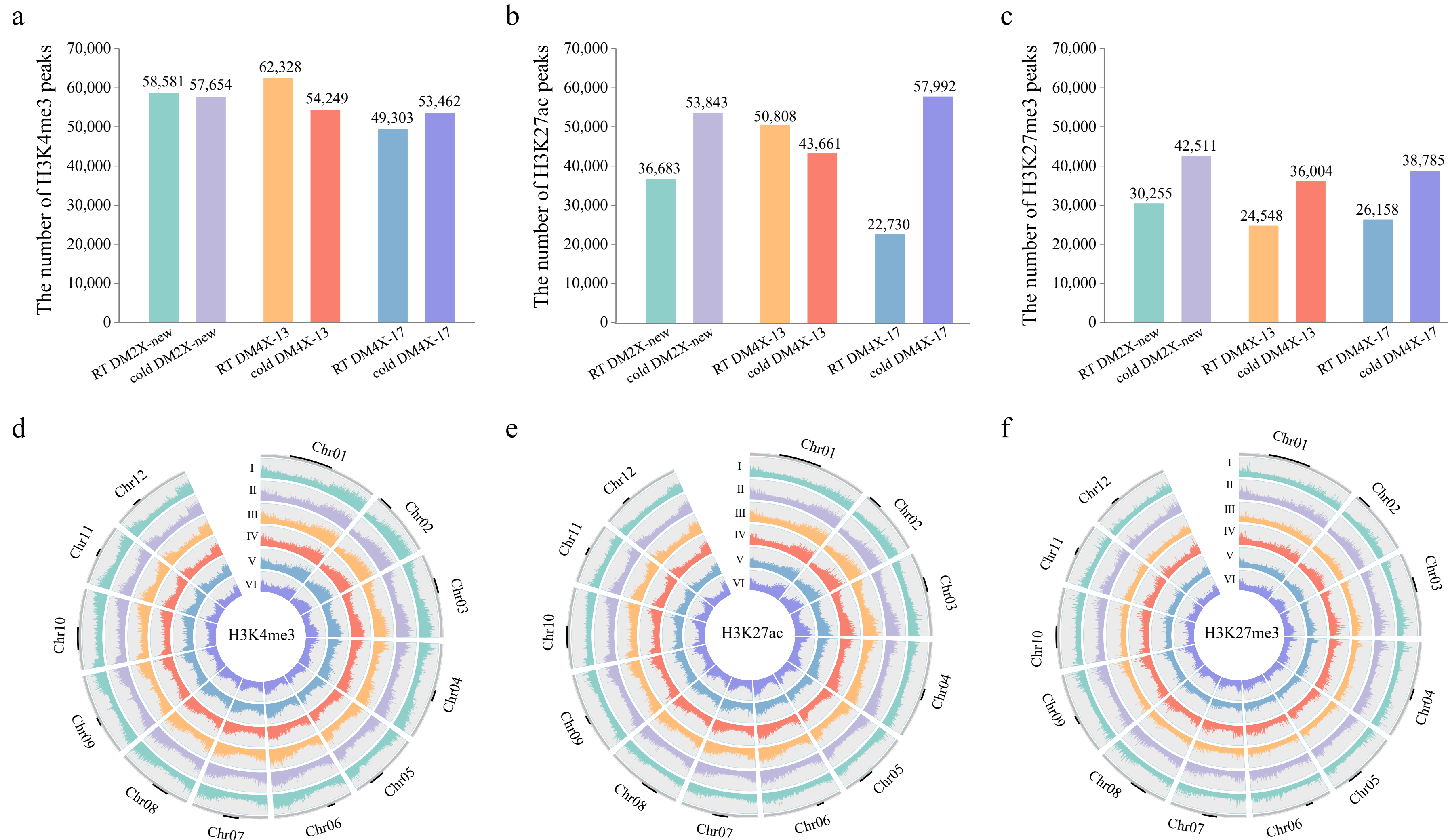

**Figure S9**

Supplement: Web_Material_uhad017 [file web_material_uhad017.zip › Figure S9.pdf]

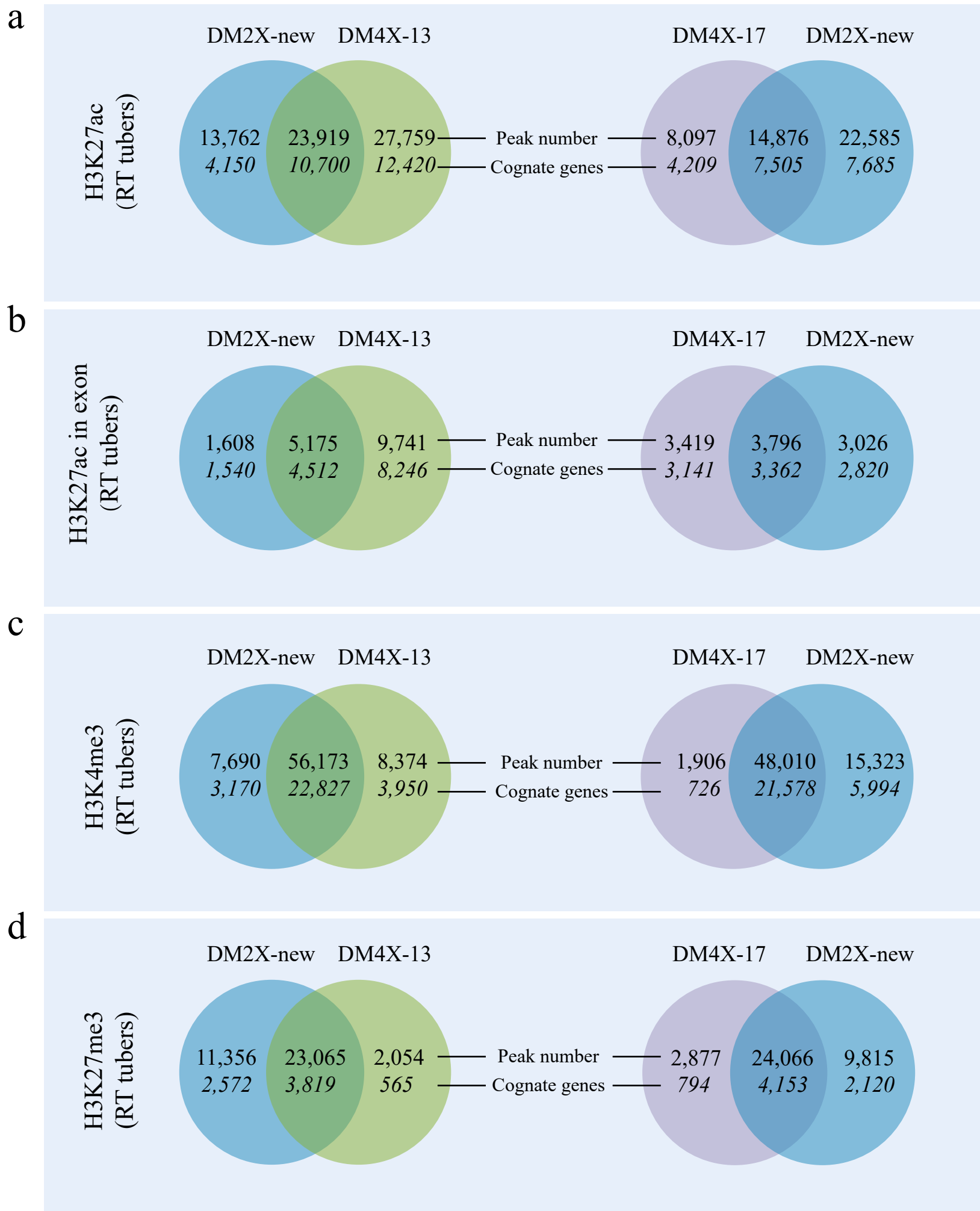

**Figure S10**

Supplement: Web_Material_uhad017 [file web_material_uhad017.zip › Figure S10.pdf]

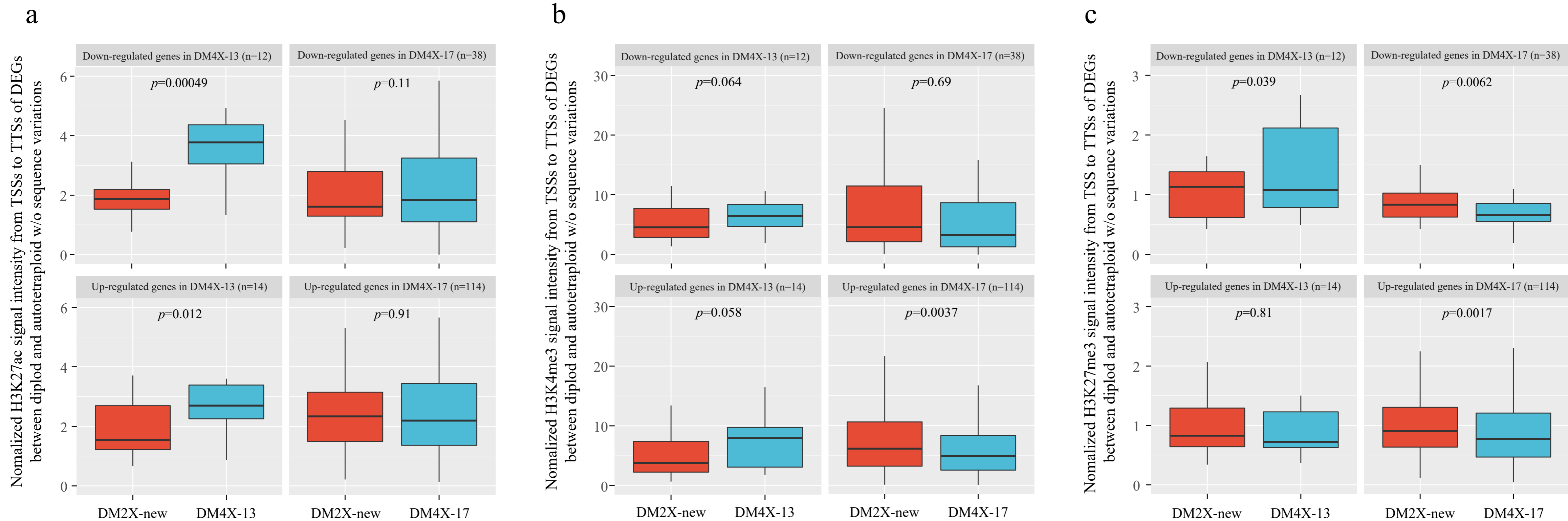

**Figure S11**

Supplement: Web_Material_uhad017 [file web_material_uhad017.zip › Figure S11.pdf]

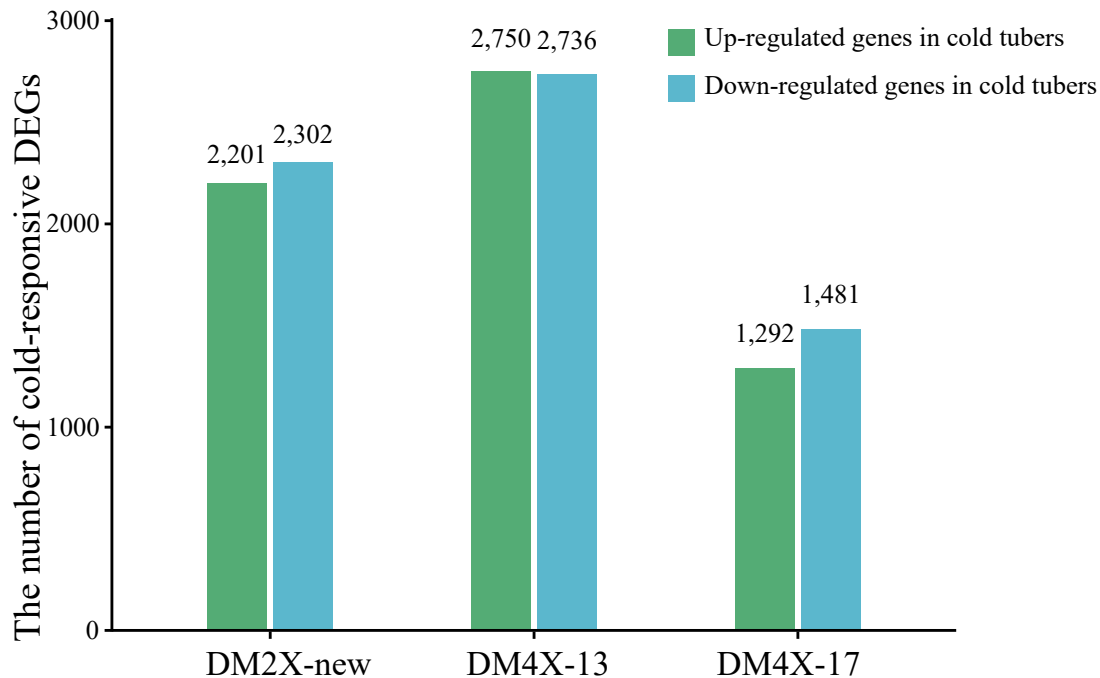

**Figure S12**

Supplement: Web_Material_uhad017 [file web_material_uhad017.zip › Figure S12.pdf]

a

GO terms of G1 (n = 900)

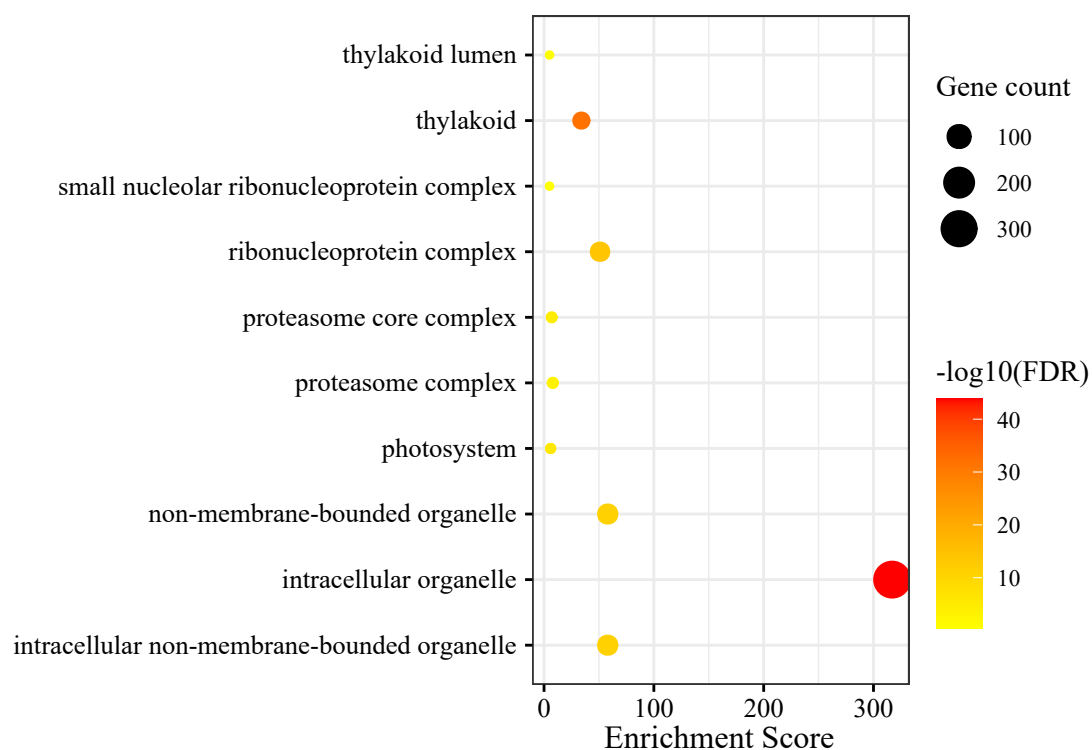

b

GO terms of G2 (n = 1,075)

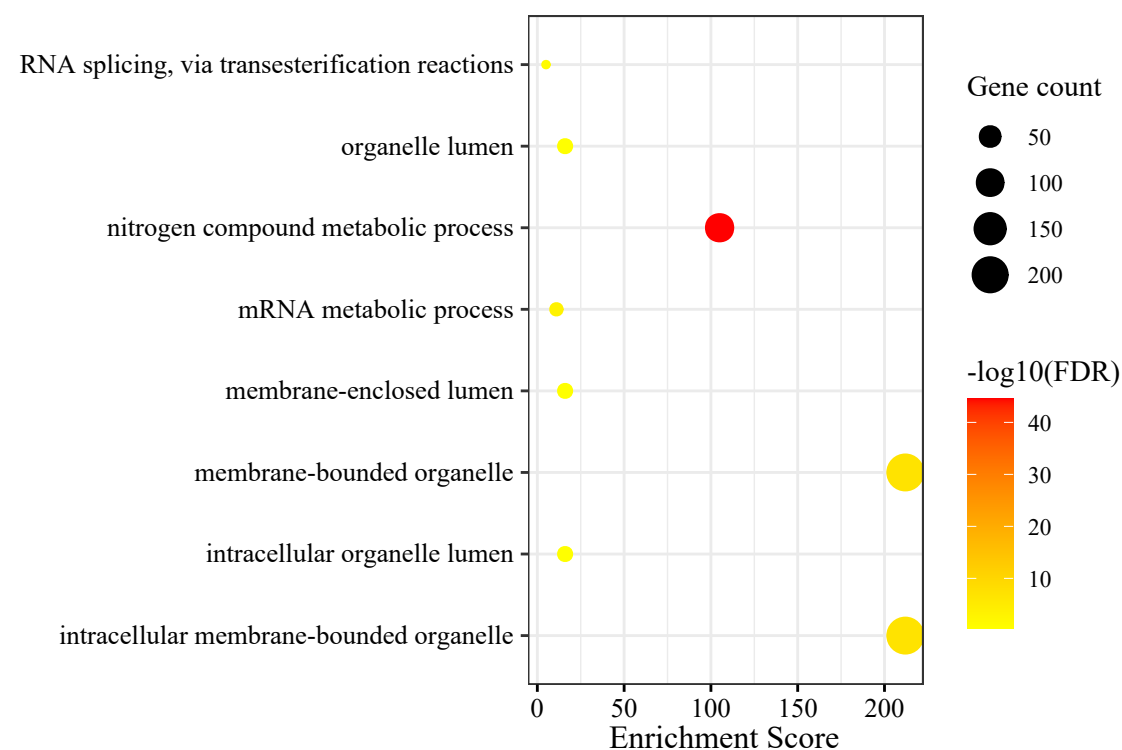

c

GO terms of G3 (n = 138)

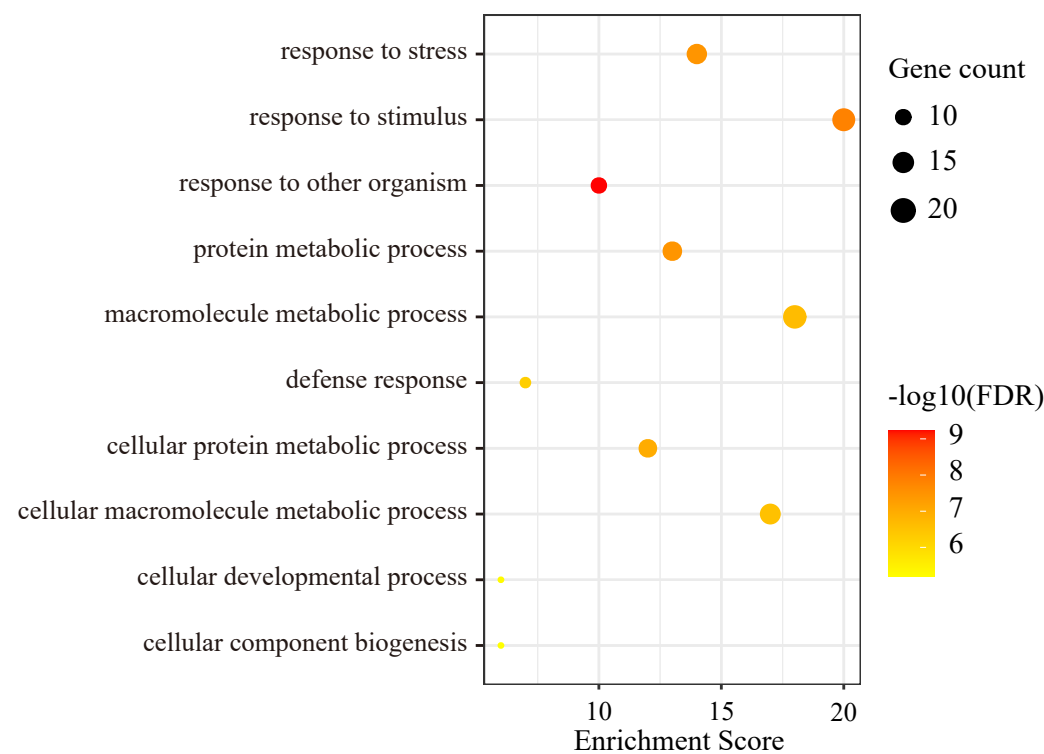

d

Go terms of G4 (n = 476)

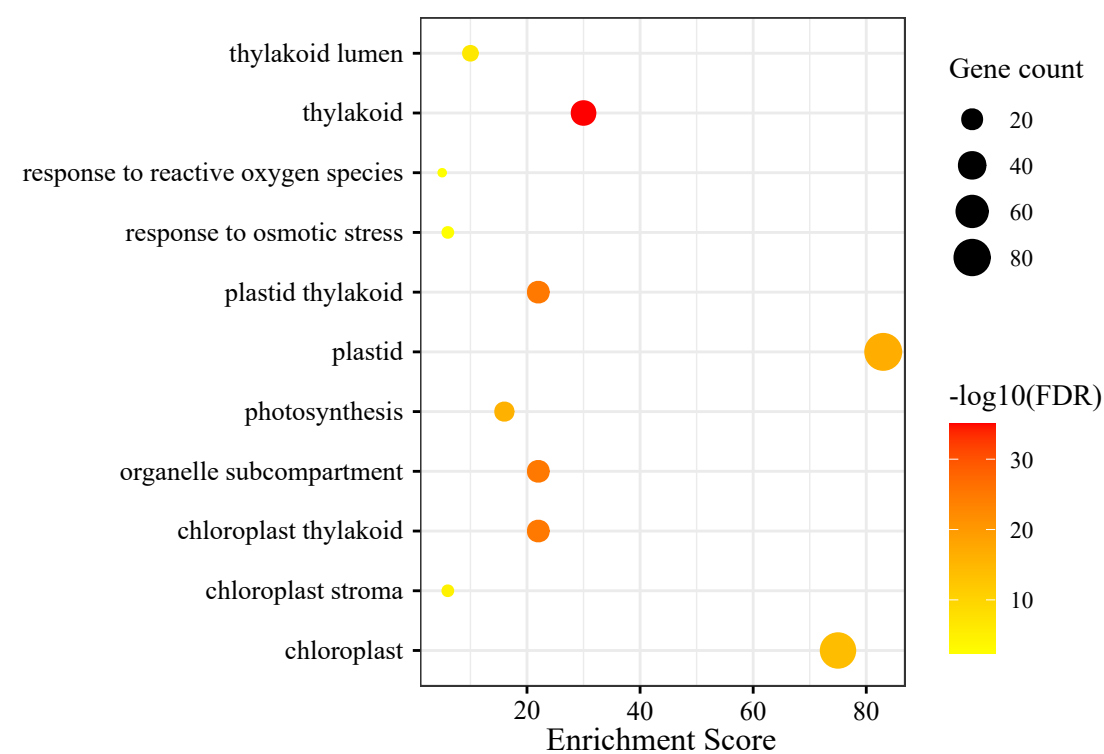

Figure S13

Supplement: Web_Material_uhad017 [file web_material_uhad017.zip › Figure S13.pdf]

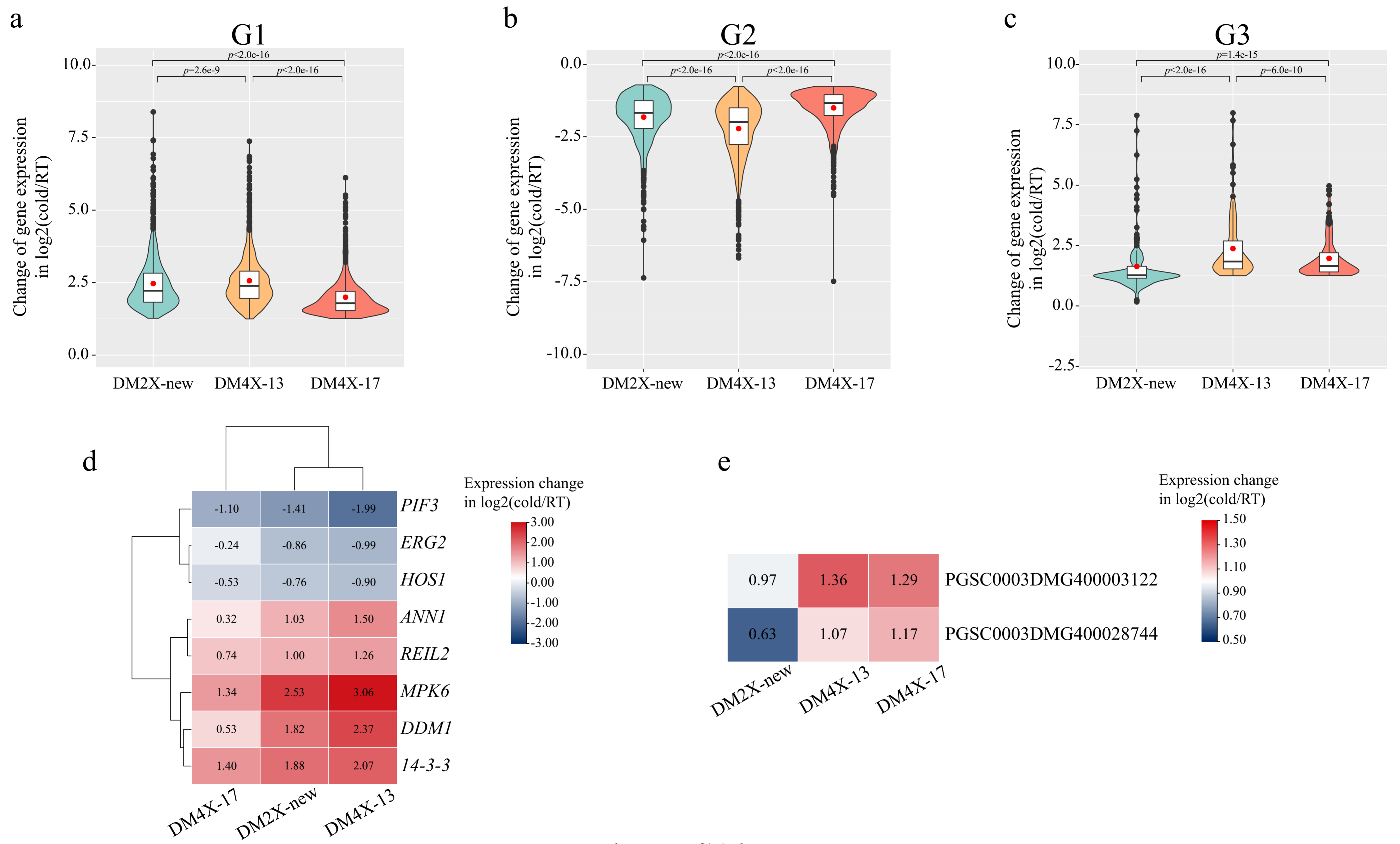

**Figure S14**

Supplement: Web_Material_uhad017 [file web_material_uhad017.zip › Figure S14.pdf]

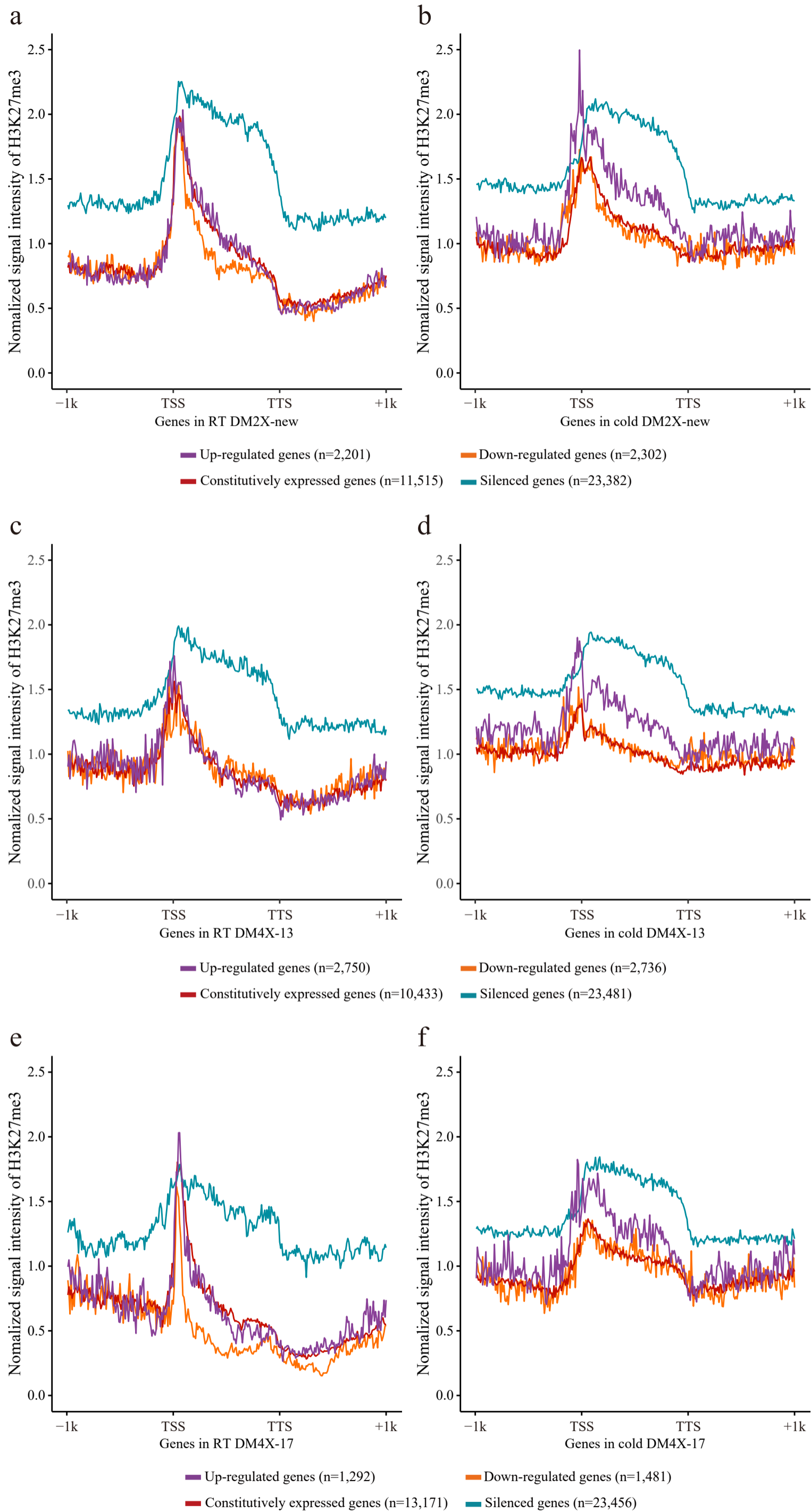

**Figure S15**

Supplement: Web_Material_uhad017 [file web_material_uhad017.zip › Figure S15.pdf]

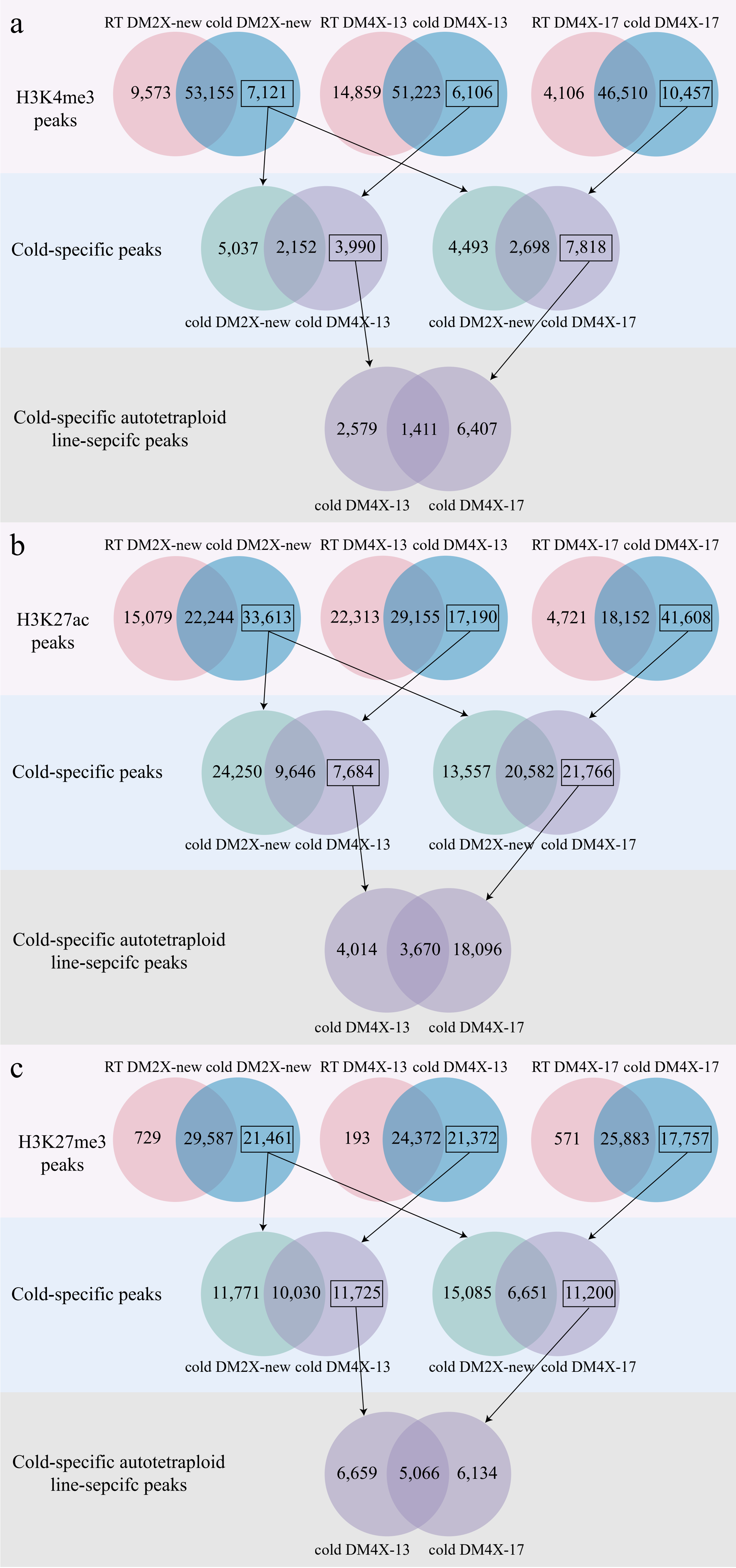

**Figure S16**

Supplement: Web_Material_uhad017 [file web_material_uhad017.zip › Figure S16.pdf]

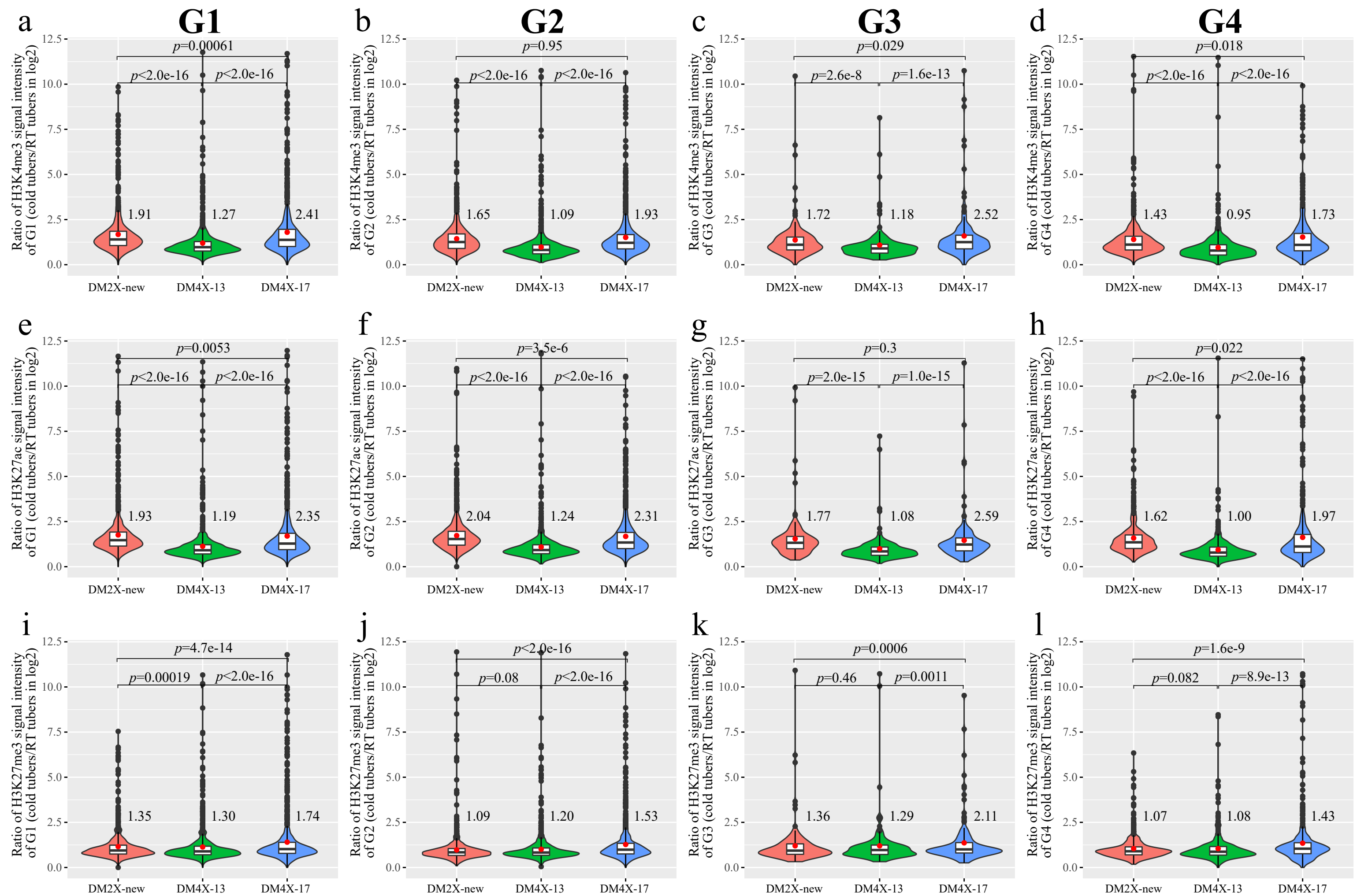

Figure S17

Supplement: Web_Material_uhad017 [file web_material_uhad017.zip › Figure S17.pdf]

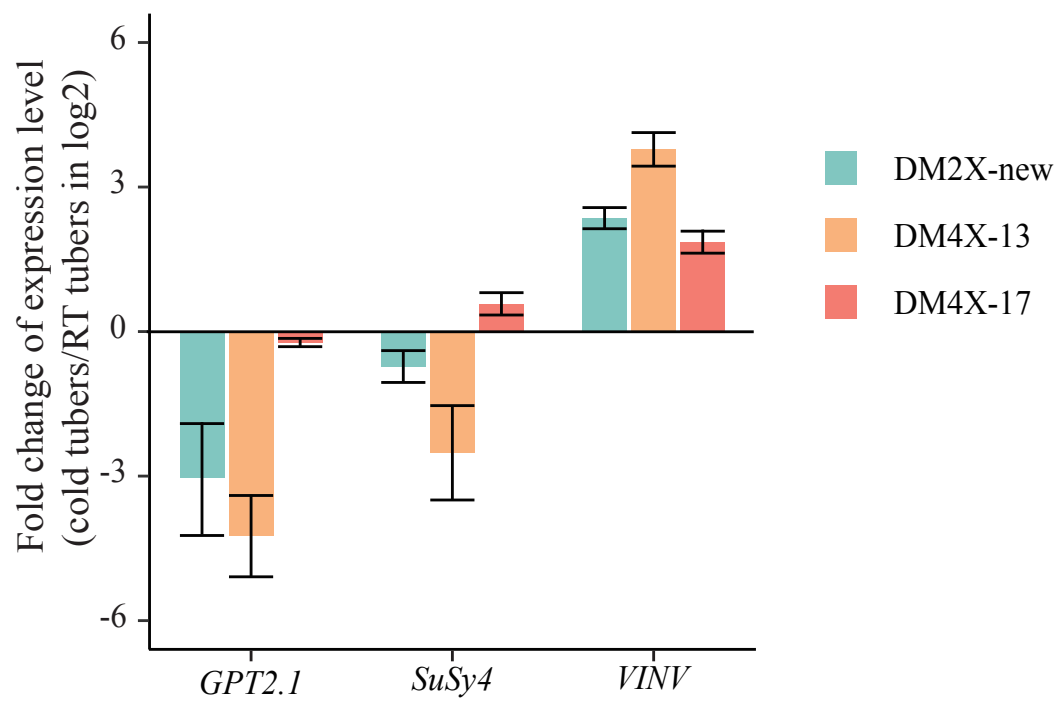

**Figure S18**

Supplement: Web_Material_uhad017 [file web_material_uhad017.zip › Figure S18.pdf]

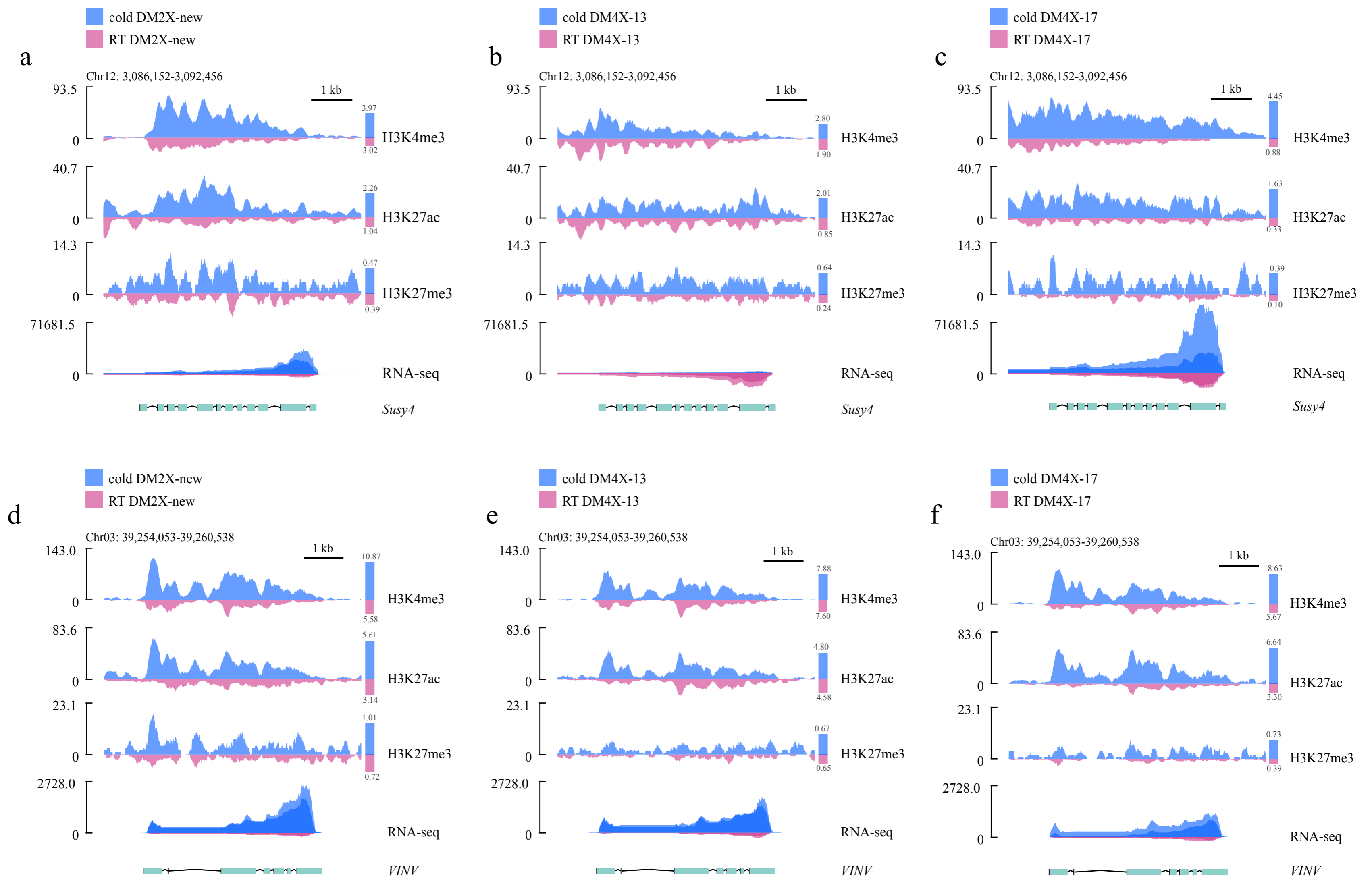

**Figure S19**

Supplement: Web_Material_uhad017 [file web_material_uhad017.zip › Figure S19.pdf]
